# Miniature bioinspired artificial compound eyes: microfabrication technologies, photodetection and applications

**DOI:** 10.3389/fbioe.2024.1342120

**Published:** 2024-02-16

**Authors:** Xian Jing, Shitao Li, Rongxin Zhu, Xiaochen Ning, Jieqiong Lin

**Affiliations:** ^1^ College of Electronic Science and Engineering, Jilin University, Changchun, China; ^2^ Jilin Provincial Key Laboratory of Micro/Nano and Ultra-precision Manufacturing, School of Mechatronic Engineering, Changchun University of Technology, Changchun, China

**Keywords:** compound eye, bionic manufacturing, microfabrication, vision imaging, microoptics

## Abstract

As an outstanding visual system for insects and crustaceans to cope with the challenges of survival, compound eye has many unique advantages, such as wide field of view, rapid response, infinite depth of field, low aberration and fast motion capture. However, the complex composition of their optical systems also presents significant challenges for manufacturing. With the continuous development of advanced materials, complex 3D manufacturing technologies and flexible electronic detectors, various ingenious and sophisticated compound eye imaging systems have been developed. This paper provides a comprehensive review on the microfabrication technologies, photoelectric detection and functional applications of miniature artificial compound eyes. Firstly, a brief introduction to the types and structural composition of compound eyes in the natural world is provided. Secondly, the 3D forming manufacturing techniques for miniature compound eyes are discussed. Subsequently, some photodetection technologies for miniature curved compound eye imaging are introduced. Lastly, with reference to the existing prototypes of functional applications for miniature compound eyes, the future development of compound eyes is prospected.

## 1 Introduction

Organisms have evolved diverse and superior visual systems, which enable them to effectively navigate, find food, communicate and avoid predators in their complex and dangerous natural environments (Smolka et al., 2011; Lim and Ben-Yakir, 2020; Casares and McGregor, 2021). Inspired by the visual systems of biological organisms in the natural world, a wide range of artificial biomimetic visual systems has been developed in the both macroscale and microscale (Liu et al., 2019; Wang et al., 2019; Ma et al., 2021). However, despite the significant advancements in size and weight, micro vision cameras still have some shortcomings, including low image quality, limited focal length, narrow field of view, and high precision requirements for detection. Compound eyes (CEs) with clusters of functional ommatidia, which are found in the insect (such as bees, flies, ants, and beetles), crustaceans (crabs, lobsters), arachnids (spiders, scorscpions), and some species of mollusks (such as certain types of snails), provide viable approaches to address the aforementioned deficiency (Somanathan et al., 2008; Somanathan et al., 2009; Alkaladi and Zeil, 2014; Morehouse et al., 2017; Arganda et al., 2020).

Compound eye consists of closely arranged ommatidia, which provide distinct advantages like a wide field of view, infinite depth of field, and sensitive response (Matsuda and Wilder, 2014; Deng et al., 2016; Hu et al., 2022). These advantages have tremendous potential applications in areas such as infrared guidance, radar warning, robot vision, medical endoscopy (Wang et al., 2021; Deng et al., 2015; Wang et al., 2022; Huang et al., 2023). The numerous advantages of compound eyes stem from their unique visual structure. Correspondingly, in order to replicate similar functionalities, artificial biomimetic compound eyes should also possess the same structural characteristics. Firstly, miniature compound eyes consist of a large number of ommatidia with individual dimension in micro/nano-scale but overall dimension probably in millimeter-scale to meet the requirements of detection range, exhibiting a cross-scale characteristic (Chen et al., 2016; Peng et al., 2016; Zhai et al., 2021). Secondly, in some cases, a single-layer planar lens array may not be able to meet the imaging precision requirements of compound eye, necessitating the combination of double or even multiple layers of planar or curved lens array to improve imaging quality, showcasing a complex 3D structure characteristic of compound eye (Chen et al., 2017; Lin et al., 2018; Ke et al., 2022). Finally, in order to meet the needs of the miniaturization of compound eye cameras, the adaptive integrated manufacturing of optical components and photoelectric sensors is also an important issue for compound eye, exhibiting a characteristic of micro-assembly (Viollet et al., 2014; Kuo et al., 2015; Xu et al., 2022). All the aforementioned characteristics pose significant challenges to the microfabrication of miniature compound eyes. With the continuous advancement of advanced materials, complex 3D manufacturing technologies, and flexible electronic detectors, a range of microfabrication technologies and photoelectric detection methods have been developed for miniature bioinspired artificial compound eyes. Scholars have gone beyond simple replication in the manufacturing of compound eyes. They have optimized the design of the structure and incorporated advanced functional materials to meet specific needs. As a result, some artificial compound eyes not only possess the advantages of natural compound eyes but also exhibit high resolution and adjustable focal capabilities similar to those of single-chambered eyes.

In this work, a comprehensive review is provided from the aspects of the microfabrication technologies, photodetection and applications of miniature compound eye. Firstly, a concise introduction is provided on the types and structural composition of compound eyes found in the natural world. Secondly, a discussion is provided on the 3D forming manufacturing techniques for miniature compound eyes. Subsequently, some photodetection approaches for diversiform artificial compound eyes are introduced. Lastly, the existing prototypes of functional applications for miniature compound eyes are listed and prospects for the future development of compound eyes are explored.

## 2 Structural composition of the compound eye

The earliest known compound eyes were discovered in the trilobites, arthropods from the Cambrian period (Land, 2005). From the birth to extinction of trilobites over billions of years, the early compound eyes have evolved along with changes in the environment. The compound eyes can be classified into two major categories based on the processing of light beams received by the ommatidia: apposition compound eyes and superposition compound eyes, catering to various visual demands (Nilsson, 1989).

### 2.1 Apposition compound eye

**FIGURE 1:**
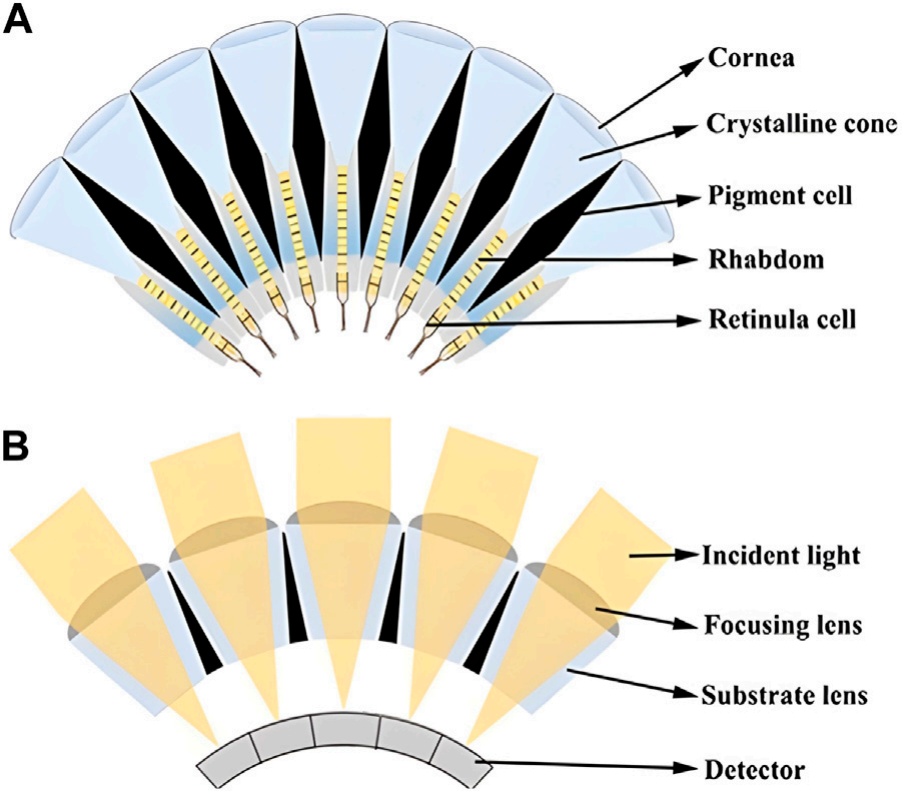
**(A)** Structure schematic diagram of the biological apposition compound eye. **(B)** Imaging schematic diagram of artificial apposition compound eye.

Apposition compound eyes consist of independent ommatidium imaging channels, which are composed of refractive devices (such as cornea and crystal cone) and a rhabdom. As light enters the refractive device, it is focused onto the rhabdom through the refractive apparatus. The rhabdom then transmits the focused light to the optic nerve for further processing and imaging. Additionally, to enhance the quality of imaging, the imaging channels of adjacent ommatidia are separated in all directions by pigment cells, preventing any interference or crosstalk of light (Guo et al., 2021). Based on the actual structure of the biological compound eye, the schematic diagram of the apposition compound eye can be simplified and depicted as shown in Figure 1A. Similarly, an imaging schematic diagram of an artificial apposition compound eye can also be obtained, as shown in Figure 1B, where the focusing lens corresponds to the cornea, the substrate lens corresponds to the crystalline cone, and the detector corresponds to the rhabdom and retinula cells.

### 2.2 Superposition compound eye

Although both the apposition compound eye and the superposition compound eye have a similar structural composition, the imaging principles are significantly different. The photoreceptive cells in superposition compound eyes receive incident light from multiple corneas, whereas the photoreceptive cells in apposition eyes receive incident light solely from the corresponding cornea.

**FIGURE 2:**
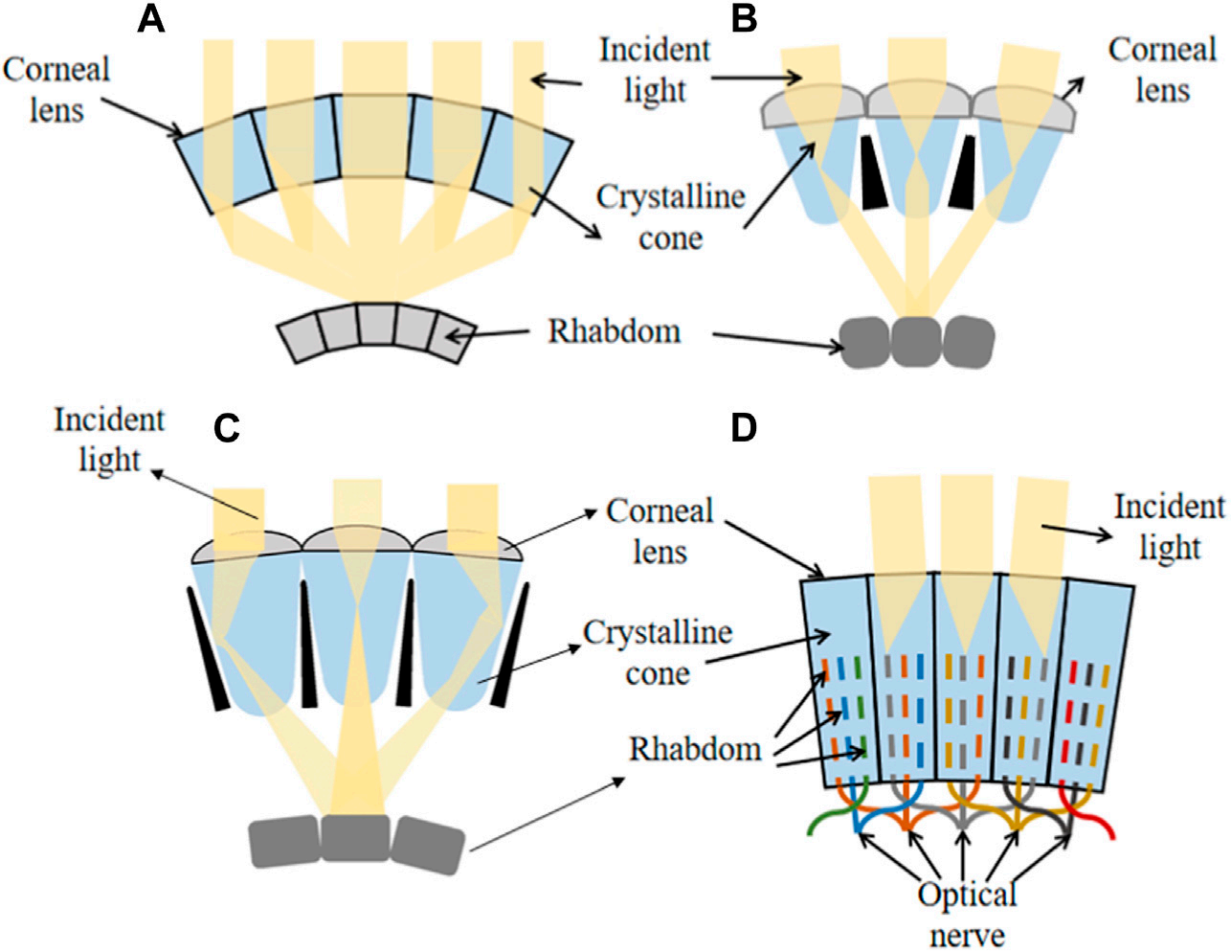
Superposition compound eyes. **(A)** Reflection superposition compound eye. **(B)** Refraction superposition compound eye. **(C)** Parabolic superimposed compound eye. **(D)** Neural superposition compound eye.

The superposition compound eye can be further categorized into optical superposition compound eyes and neural superposition compound eyes, depending on the different mechanisms of light perception. In optical superposition compound eyes, incident light is focused onto a specific region of photoreceptive cells through optical reflection or refraction. As a result, optical superposition compound eyes exhibit higher sensitivity and smaller focal ratios (F-numbers) compared to apposition compound eyes (Land et al., 1979). Figure 2 illustrates three types of optical compound eyes with superposition, namely, optical refraction superposition, optical reflection superposition, and parabolic superposition, corresponding to optical propagation paths generated by the mechanisms of refraction, reflection, and a combination of both, respectively (Land, 1981; Wagner et al., 2022). Organisms with optical superposition compound eyes inhabit dark environments and possess remarkable light-capturing capabilities and efficient utilization of light energy (Frederiksen and Warrant, 2008; Song et al., 2017). Whereas the neural superposition compound eye receives incident light through the photoreceptive nerve from multiple ommatidia. Therefore, when the number of ommatidium is the same, the neural superposition compound eye has higher resolution.

## 3 Microfabrication technologies for artificial compound eye

These characteristics of functional components in micro systems present significant challenges to existing microfabrication technologies. A variety of advanced technologies and materials have been employed in the manufacturing of micro compound eyes. The following is a review based on the different manufacturing technologies for planar compound eyes and curved compound eyes.

### 3.1 Microfabrication technologies for planar compound eye

Prior to the 21st century, manufacturing artificial compound eyes primarily concentrated on flat compound eyes due to the limitations of processing technology during that period (Oikawa et al., 1981; Borrelli et al., 1985; Popovic et al., 1988; Cheng et al., 2019). Various technologies, including stamping, reversal replication, droplet deposition, thermal reflow and lithography, were employed to enhance the precision and efficiency of manufacturing planar compound eyes (Yang et al., 2005; Hong et al., 2006; Chang et al., 2007; Pan and Su, 2008; Yu et al., 2013; Cherng and Su, 2014; Park et al., 2014; Yong et al., 2015; Grigaliunas et al., 2016). Among these technologies, the processing methods for planar compound eyes can be roughly divided into two categories: direct forming technology and indirect forming technology. Unlike direct forming technology, which directly deals with the contour of compound eyes using one or more specialized manufacturing technique, indirect forming technology involves initially producing the mold of the compound eyes and then obtaining the planar compound eye structure through demolding or hot embossing processes.

#### 3.1.1 Direct forming technology

Direct forming technology for planar compound eyes primarily relies on micro-droplet manufacturing and femtosecond laser processing techniques. Micro inkjet printing is a commonly used micro-droplet manufacturing technology, widely applied in fabricating microstructures for electronic communication devices, micro-optic systems, and optical interconnections (Cox et al., 1996; Cox et al., 2001; Lee et al., 2010; Yu et al., 2013). Micro inkjet printing technology allows for speedy prototyping over large areas, making it highly efficient for microlens preparation. Furthermore, it provides a simple and rapid approach to microlens processing.

**FIGURE 3:**
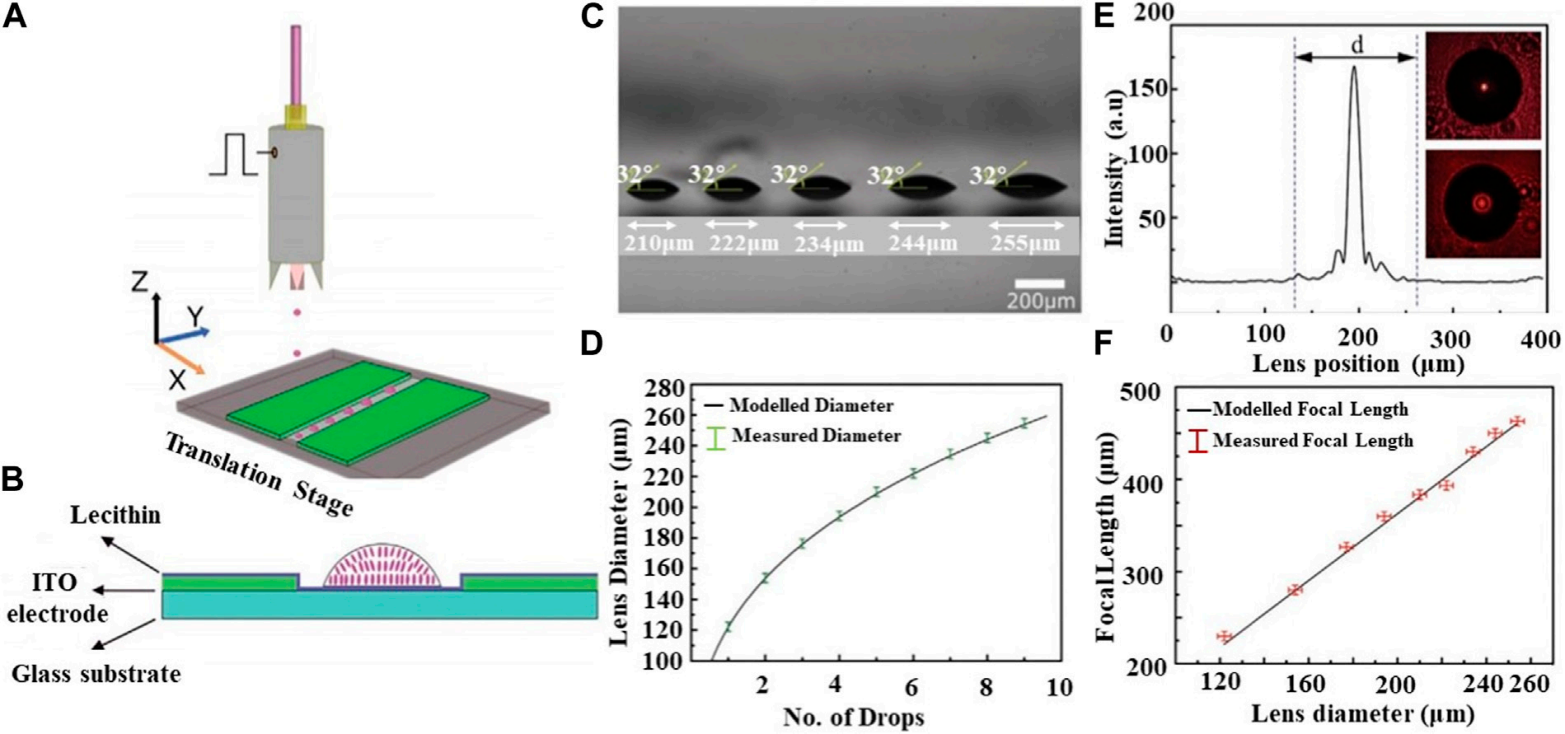
The schematic diagram of processing and change curves of microlenses (Reproduced with permission from (Kamal et al., 2020)). **(A)** Schematic of the fabrication process. **(B)** A cross-section of a glass substrate with in-plane electrodes and a single microlens. **(C)** Image of five microlenses with different diameters and same contact angles. **(D)** The relationship between lens diameter and number of drops. **(E)** Intensity of 255 μm diameter lens at the image plane with CCD images at insets. **(F)** The relationship between focal length and lens position.

Inkjet printing offers the advantage of flexibility, allowing for the easy fabrication of microlens arrays with various curvatures and sizes. E. Parry et al. demonstrated the tunability of the lens focal lengths by adjusting the temperature (Parry et al., 2017). While the inkjet printing process enables the quick fabrication of a large-area microlens array, it is necessary to combine it with voltage-tunable focal length technology in order to fully utilize the capability of adjusting the focal length. W. Kamal et al. proposed the use of inkjet printing to fabricate thermally tunable liquid crystal (LC) microlenses. The inkjet printing process is illustrated in Figure 3A. A Lecithin-coated substrate at 20°C was employed to promote a plano-convex lens configuration with a homeotropic alignment of the LC director. Indium-tin-oxide (ITO) was deposited on the glass substrate using the multiple droplet deposition technique. The cross-section of the glass substrate with in-plane electrodes and a single microlens is shown in Figure 3B. Multiple droplets were deposited at the same location on the glass to increase the microlens volume. Figure 3C displays five microlenses with diameters ranging from 210–255 μm. The relationship between lens diameter and number of droplets is depicted in Figure 3D, clearly indicating that the lens diameter is governed by the drop volume and aligns well with the modeled drop diameter for a 32° contact angle spherical cap. The light intensity profile of the focused spot from a 255 µm diameter microlens is shown in Figure 3E. Figure 3F emphasizes that the focal length of the microlenses, dependent on the radius of curvature, changes with different voltages. It is showed an electrical tunability of microlenses on the in-plane electrodes (Kamal et al., 2020). In addition, V. Vespini et al. proposed a forward pyro-electrohydrodynamic inkjet printing system that utilizes a temperature gradient to address the issue of limited working distance in a contact-free mode (Grimaldi et al., 2012; Grimaldi et al., 2013; Vespini et al., 2016). This system is capable of printing and transferring liquid shots and pixels with defined dimensions, achieving high resolution even over long distances. Meanwhile, the fabrication of microlenses in a tunable multiscale range is an important issue. Vespini et al. proposed an electrohydrodynamic assembly method to achieve this goal (i.e., between 25 and 200 μm diameter) Vespini et al. (2015).

Femtosecond laser processing is currently widely regarded as one of the most promising micro/nano processing technologies due to its ability to prepare small-sized and high-precision structures. It offers advantages such as short processing cycles and high instantaneous power, enabling the processing of high-hardness materials. Femtosecond laser processing can be classified into two main categories: femtosecond laser modification (Pan et al., 2014; Deng et al., 2015; Wei et al., 2018) and femtosecond laser two-photon polymerization technology (Guo et al., 2006).

Femtosecond laser modification technology utilizes a point-by-point processing method, which typically results in rough processing quality and low imaging quality of optical components. To enhance the image quality of optical lenses following femtosecond laser modification, the commonly used approach involves the utilization of wet etching technology to reduce surface roughness. This etching process exhibits selectivity, occurring exclusively within the region subjected to laser irradiation. This directional material removal enables the formation of high-quality concave microlens arrays (Liu et al., 2020). This method involves using femtosecond laser to induce the formation of modified regions on the surface, followed by the application of hydrofluoric acid to, etch the modified region, thereby accelerating the formation of microlenses (Chen et al., 2014).

In the study conducted by F. Chen et al., femtosecond laser ablation combined with wet etching was employed to fabricate both rectangular and hexagonal concave microlens arrays. The process involved three steps: firstly, ablation-induced craters with diameters of a few micrometers were created on polished silica glass chips. Subsequently, the samples with craters were treated in a 5% hydrofluoric (HF) acid solution with the assistance of an ultrasonic bath at 23°C. During this process, the chemical etching velocity was accelerated in the laser-induced craters, leading to the formation of concave spherical surfaces. The fabrication of the microlens arrays was completed in tens of minutes. Finally, the samples were cleaned using an ultrasonic bath in acetone, alcohol, and deionized water for 15 min each, followed by drying in ambient air (Chen et al., 2010).

On certain intricate structures, femtosecond laser two-photon polymerization technology has the capability to achieve precise point-by-point processing (Kawata et al., 2001; Tian et al., 2015) proposed a microlens array with differently curved unit lenses known as MLADC (Microlens Array with Different Curvature). The process involved spin-coating SU-8 photoresist onto a glass slide that had been cleaned with acetone and anhydrous ethanol. The lenses were then processed using femtosecond laser pulses. The resulting microlenses were placed in a developer solution to eliminate any unpolymerized photoresist, leading to the complete structure of the compound eye lens (Tian et al., 2015).

#### 3.1.2 Indirect forming technology

In the planar compound eye processing discussed in this paper, the method used for replicating and embossing the compound eye model can be described as indirect molding technology. Specifically, in the replication method for the planar compound eye model, a polymer replication process has been employed for the mass production of a microlens array due to its cost effectiveness (Hocheng et al., 2008; Chang and Tsai, 2015; Chakrabarti et al., 2016; Chen et al., 2016). However, the use of a polymer microlens array is limited when it comes to applications in high optical, thermochemical, or mechanical environments. To address this limitation, researchers have discovered that a glass microlens array possesses excellent properties such as acid, moisture, and wear resistance. Glass microlens arrays can be fabricated through chemical etching and mechanical machining. However, mechanical machining is not only inefficient but also makes it challenging to achieve high quality results at a low cost.

**FIGURE 4:**
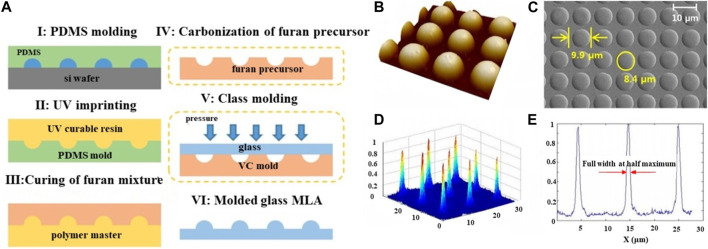
The schematic diagram of processing and measurement results of microlenses (Reproduced with permission from (Kim et al., 2018)). **(A)** Schematic of the fabrication process. **(B)** AFM and **(C)** SEM measurement results of glass molded microlens array. **(D)** 3D intensity profile and **(E)** cross-sectional profile of the focused laser beam of the glass molded microlens array.

In order to enhance processing efficiency and achieve a high-quality glass microlens array, Y.K. Kim et al. proposed a glass molding technology to replicate the mold shape onto a glass substrate. The process for manufacturing the microlens array is illustrated in Figure 4A. Firstly, a microlens array master pattern was created using a thermal reflow process. A polymer master was then obtained by a double-replication process involving PDMS molding and UV imprinting, which served to protect the master pattern. Next, a mixture of furan resin, initiator, and organic solvent was poured onto the polymer master, resulting in the formation of a furan precursor after thermal curing. By carbonizing the curved furan precursor, a vitreous carbon mold was obtained. Both the carbonization of the furan precursor and the glass molding process were carried out in a vacuum with heating. Finally, using the motor-driven pressure, the glass microlens array was formed using the vitreous carbon mold. The AFM and SEM measurements in Figures 4B, C reveal that the glass molded microlens array possesses a uniform and high-quality surface. This confirms the success of the glass molding replication process in manufacturing the microlens array with a high surface quality. Additionally, Figure 4C demonstrates the high precision of the glass molded microlens, with a diameter of 8.4 μm, pitch of 9.9 μm, and sag height of 0.699 μm. To examine the optical properties of the fabricated microlens array, the energy intensity distribution was measured. Figures 4D, E displays the 3D intensity profile and cross-section profile, revealing uniform light intensity in the microlens spots (Kim et al., 2018).

**FIGURE 5:**
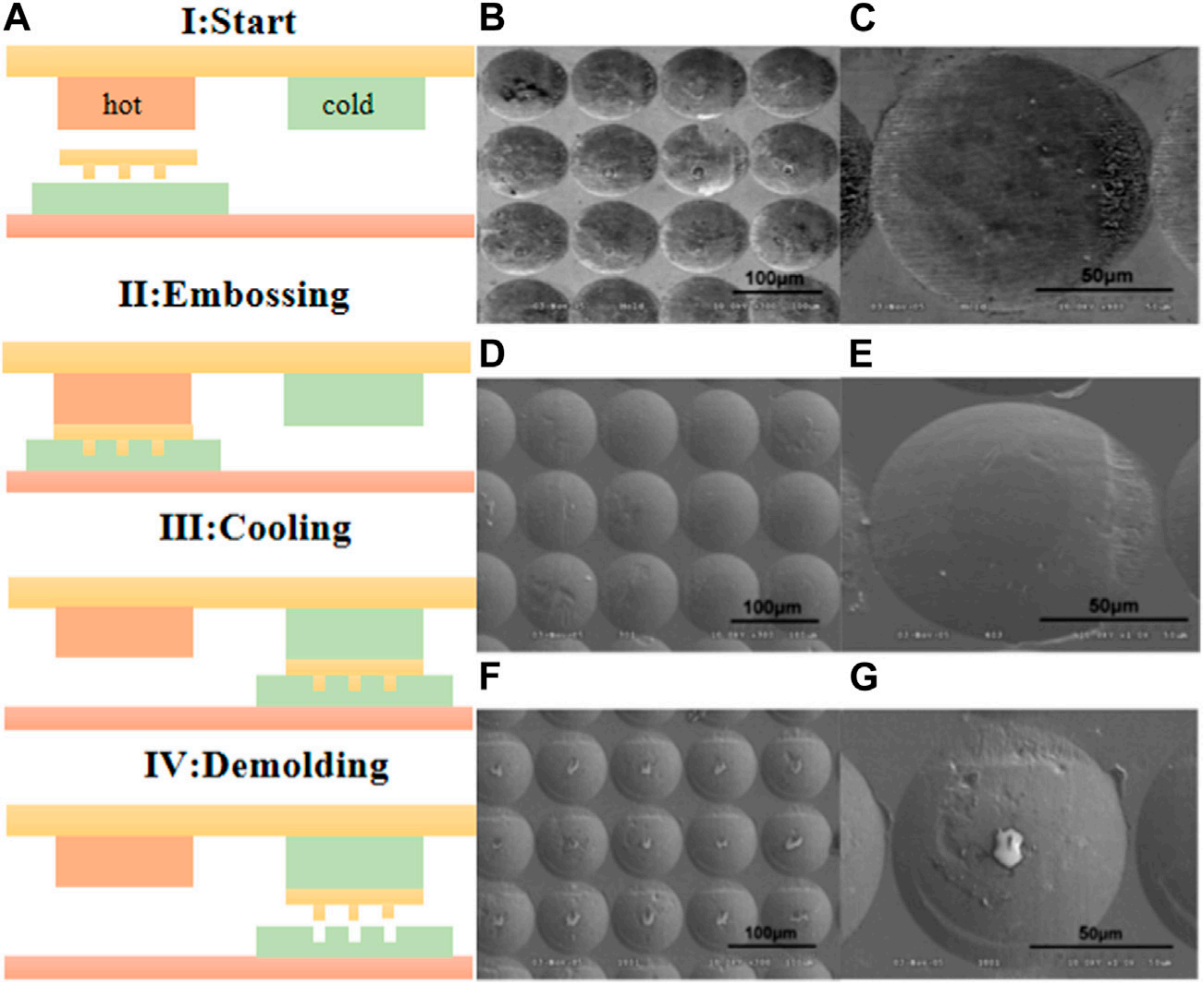
Embossing technology (Reproduced with permission from (Yao et al., 2010)). **(A)** Schematic of the fabrication process. **(B)** The embossing master and **(C)** partial enlarge image. **(D, E)** The image of embossed copolymer surface. **(F)** The embossed microlenses after contact heating 10 s and **(G)** partial enlarge image.

The embossing process typically involves several consecutive stages, which require thermal cycling. However, the large thermal mass of the embossing tool leads to long cycle times, exceeding 10 min. Prolonged exposure to elevated temperatures can result in polymer degradation. To address this issue, D. G. Yao et al. proposed a sequential heating and cooling embossing strategy to reduce cycle times in hot embossing of polymer microstructures Yao et al. (2010). The embossing process for manufacturing microlens arrays is illustrated in Figure 5A. The polymer is heated and softened at 250°C using a hot station, and then transferred to a cold station for cooling until reaching the mold opening temperature. This significantly reduces the cycle time of the hot-pressed microstructure, with heating time less than 3 s and a total cycle time of approximately 10 s. SEM images of the microlenses are presented in Figures 5B–E, demonstrating good replication fidelity, including sharp edge replication and minimal mold surface defects. However, after a 10-s contact heating time, some detached residuals were observed at the center of the embossed microlenses, as depicted in Figures 5F, G. This suggests that prolonged embossing and high temperatures can lead to excessive adhesion, emphasizing the importance of strict control over factors such as pressure, temperature, and time during the fabrication process. Surface defects are prone to develop, which can compromise the shape accuracy of the microlens.

A comparison of microfabrication technologies for miniature planar compound eyes is presented in Table 1. The technologies are evaluated based on processing efficiency, surface quality, and the reported diameter of the ommatidium (*d*_*o*_). Inkjet printing enables direct droplet deposition on flexible or rigid substrates. The volume and arrangement of droplets can be controlled by adjusting the pulse duration and amplitude of the electrical actuation. However, deformation may occur after the solidification of photosensitive materials, resulting in certain deformations. The femtosecond laser-enhanced wet etching method utilizes femtosecond laser irradiation to modify the pit array on the surface. Hydrofluoric acid is then used to, etch the modified region, thus accelerating the formation of microlenses. This method is suitable for processing hard materials, offering good stability and surface quality. Femtosecond laser two-photon polymerization technology allows for true three-dimensional processing with high accuracy. However, the point-to-point processing approach used in this method results in lower processing efficiency. Glass molding replication technology is a cost-effective method for producing microlens arrays. It allows for the mass production of large-area microlenses with high precision. The process can be repeated multiple times to produce microlenses over a large area. While this technology offers high processing efficiency, achieving a completely smooth surface can be challenging due to material adhesion during replication. Embossing technology performs similarly to glass molding replication. It also offers high machining efficiency, but the surface quality can be affected by material adhesion.

**TABLE 1:** Microfabrication technologies for miniature planar compound eye.

Fabrication technology		Processing efficiency	Surface quality	Reported *d*_*o*_
Direct forming technology	Inkjet printing	Medium	High	30 μm (Yu et al., 2013)
	Femtosecond laser-enhanced wet etching method	Medium	High	30.54 μm (Chen et al., 2010)
	Femtosecond laser two-photon polymerization technology	Low	High	∼20 μm (Tian et al., 2015)
Indirect forming technology	Glass molding replication technology	High	Medium	8.4 μm (Kim et al., 2018)
	Hot embossing technology	High	Medium	100 μm (Yao et al., 2010)

### 3.2 Microfabrication technologies for curved compound eye

Although the planar compound eye has significantly enhanced the imaging clarity of optical systems, its planar arrangement restricts its applications in various fields. To address these limitations, a curved structure has been explored as a potential solution. Researchers are now shifting their focus from planar compound eyes to curved compound eyes in order to achieve larger field of view, higher integration, and intelligence (Maddern and Wyeth, 2008; Li and Yi, 2010; Deng et al., 2016; Liang et al., 2019). The advancement of micro-nano processing technology and flexible materials has led to significant progress in improving the resolution, field of view, antifouling ability, and zoom integration of curved compound eyes. This section aims to discuss the curved compound eye under different processing types, including single manufacturing technologies such as femtosecond laser two-photon polymerization, as well as other composite manufacturing methods. The objective is to explore the various advancements and achievements in this field.

**FIGURE 6:**
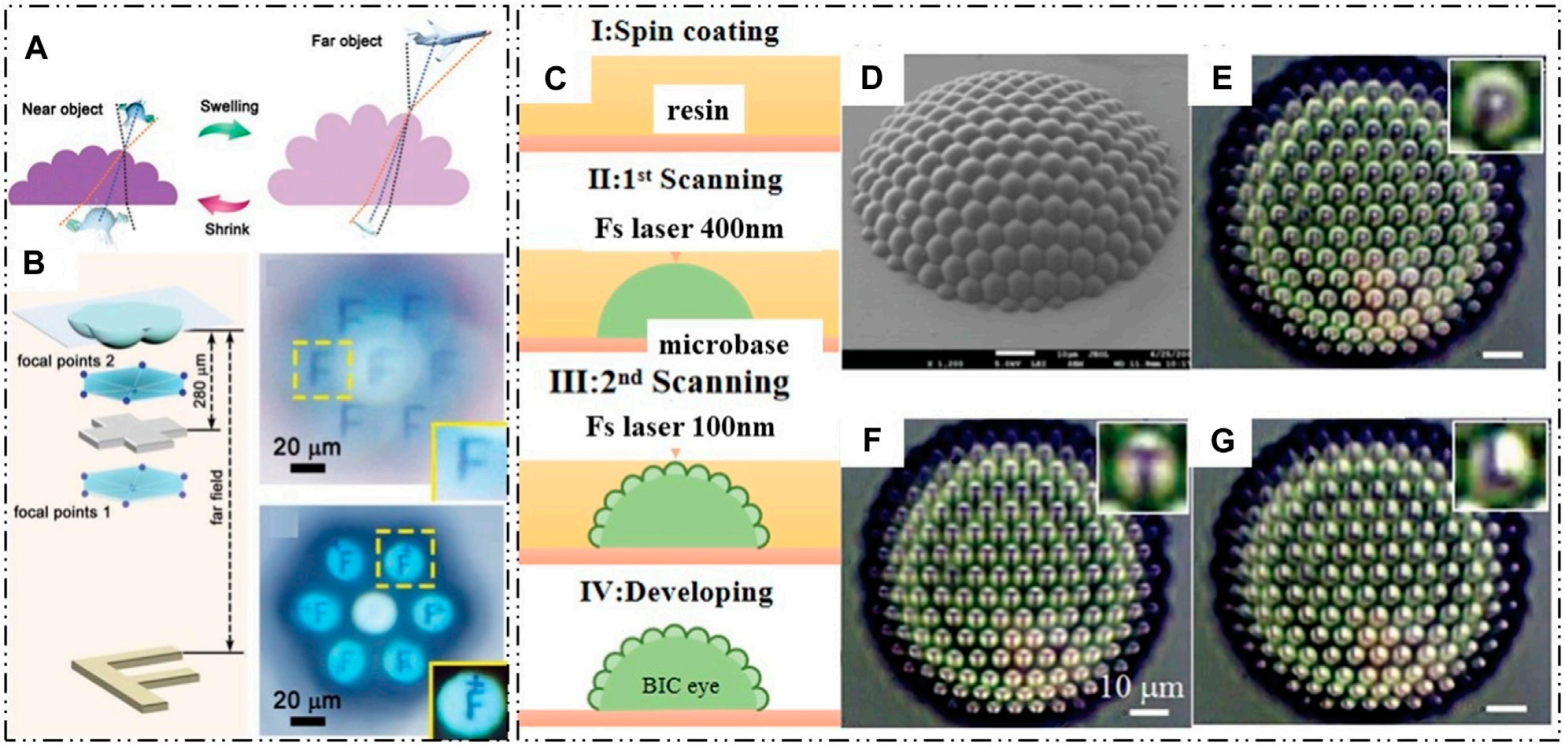
Fabrication of compound eyes using femtosecond laser two-photon polymerization technology. (Reproduced with permission from (Wu et al., 2014; Ma et al., 2019)). **(A)** The contraction and expansion of BSA. **(B)** Zoom and imaging of BSA compound eye. **(C)** Schematic of the fabrication process. **(D)** SEM of the artificial compound eye. **(E**–**G)** The imaging of the letters “P”, “T” and “L”.

In previous research on natural compound eyes and artificial compound eyes, it has been observed that both types have ommatidia with fixed focal lengths, limiting their ability to achieve variable focal length imaging. To address this limitation, Z.C. Ma et al. developed an intelligently tunable compound eye lens. They fabricated a stimuli-responsive compound eye based on bovine serum albumin (BSA) using femtosecond laser two-photon polymerization. The compound eye lens consists of a double-layer structure. The outer layer is created using two-photon polymerization technology to drop BSA aqueous solution gel onto a glass substrate, while the inner layer is made using the same processing method but with SU-8 photoresist solution. Figure 6A illustrates the contraction and expansion of BSA at different pH values, demonstrating that not only can the focal length of the compound eye lens be adjusted, but also the field of view (FOV) can be modified within the range of 35°–80°. Furthermore, Figure 6B presents the imaging quality and imaging distance of the compound eye under different pH values (Ma et al., 2019). These findings showcase the potential of intelligently tunable compound eyes in achieving variable focal length and adjusting the FOV.

Although femtosecond laser two-photon polymerization technology has been used to fabricate 3D high-precision compound eye lenses, the processing efficiency is hindered by the point-to-point processing method. To overcome this challenge, D. Wu et al. proposed a scanning strategy to control the voxel size and introduced a high-speed voxel-modulated femtosecond laser direct writing technology to achieve the three-dimensional structure of artificial compound eyes. This advancement not only enables rapid processing of artificial compound eyes but also realizes a small (84 μm) wide-field imaging system without distortions. The artificial compound eye was fabricated using laser direct writing, as shown in Figures 6C, D. The first step involved scanning the inner macrobase with a 400 nm fs laser, followed by the scanning of small ommatidia on the macrobase using a 100 nm fs laser without any gap. While the small voxel mode allows for highly precise devices, it is limited by prolonged fabrication times, which can make this method impractical. However, an improved laser direct writing strategy has been developed, achieving a large aperture (0.4), a high fill factor (100%), and ultra-low surface roughness. The images of the letters in Figures 6E–G demonstrate that the artificial compound eye exhibits high optical imaging quality and uniformity in all directions (Wu et al., 2014).

**FIGURE 7:**
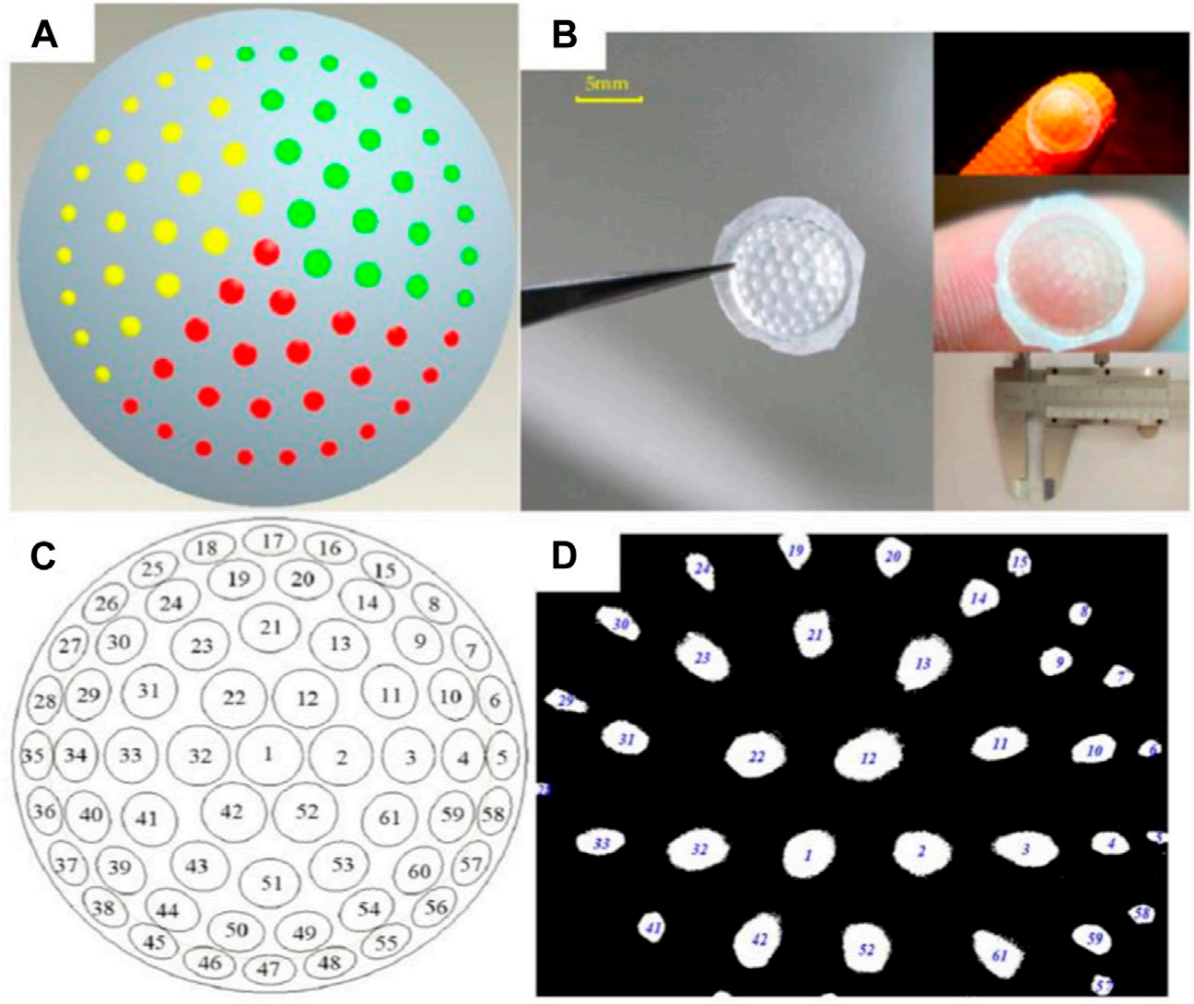
Ultra-precision machining of flexible zoom compound eye. [Reproduced with permission from (Li et al., 2018)]. **(A)** Flexible zoom compound eye distribution map. **(B)** PDMS compound eye model. **(C)** Flexible zoom compound eye positioning map. **(D)** Flexible zoom compound eye imaging.

Compound processing involves creating a curved compound eye using two or more methods. Mechanical deformation, imprinting, vacuum absorption, and mold rotation are commonly employed techniques. These methods are straightforward and efficient. In the field of zoom imaging for curved compound eyes, L. Li et al. proposed a design for a flexible zoom artificial compound eye Li et al. (2018). The surface compound eye is divided into three fan-shaped regions with different focal lengths, as depicted in Figure 7A. Through aspheric optimization, the spherical aberration of each region is reduced to 1% of the initial value. The design not only allows for focal length adjustment but also addresses the issue of poor imaging quality at the edges of curved compound eyes. The structure of the artificial curved compound eye is formed using mold processing. The mold is created using a precision five-axis CNC machine, and the molding material used is polydimethylsiloxane (PDMS). A mixture of curing agent and PDMS, in a ratio of 1:10, is poured into the mold and cured at a fixed temperature of 80°C for 1 h to obtain the complete structure shown in Figure 7B. The imaging effect of the compound eye and a multi-eye positioning experiment are demonstrated in Figures 7C, D.

**FIGURE 8:**
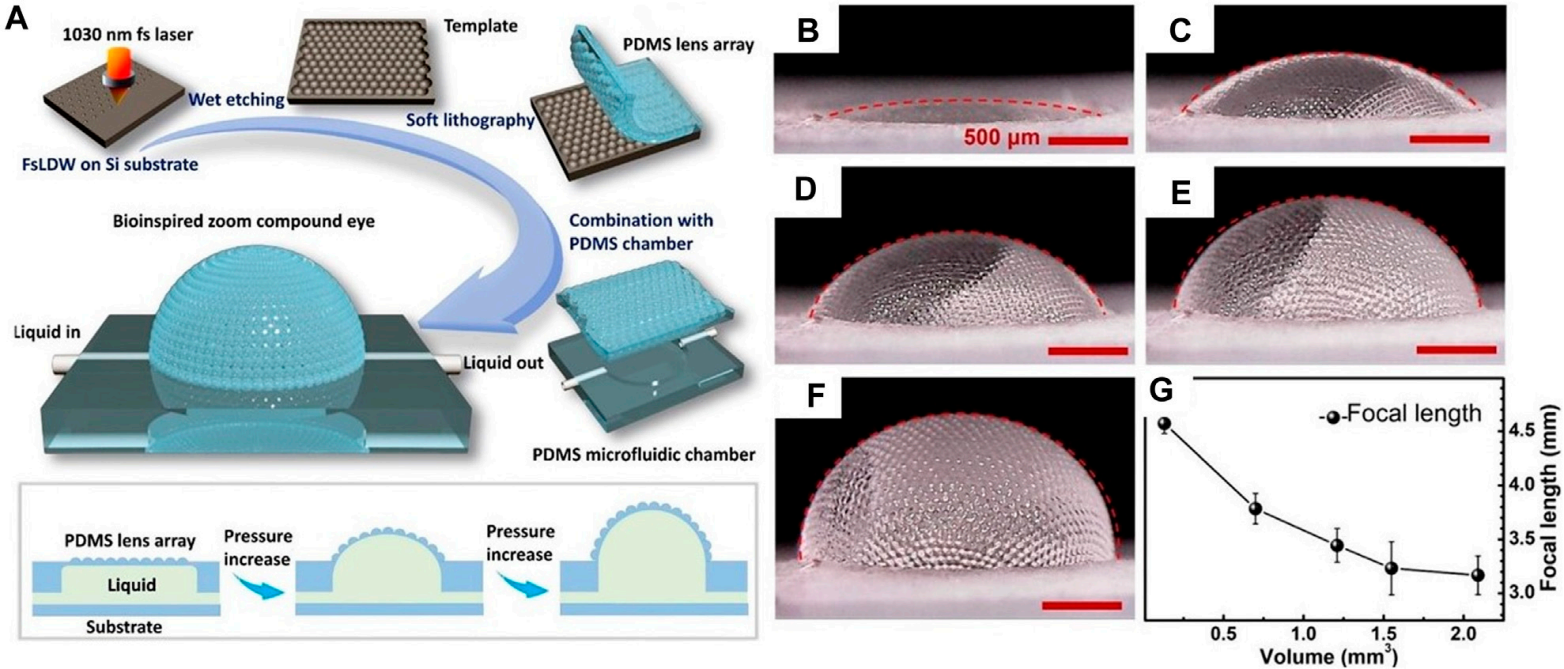
Fabrication of zoom compound eye using femtosecond processing wet-etching metho. (Reproduced with permission from (Cao et al., 2020)). **(A)** Processing flow and working principle of tunable bionic compound eye. **(B–F)** Compound eye zoom under different liquid volumes, **(B)** 0.13 cubic millimeter, **(C)** 0.7 cubic millimeter, **(D)** 1.21 cubic millimeter, **(E)** 1.55 cubic millimeter and **(F)** 2.09 cubic millimeter. **(G)** Changes of liquid volume and compound eye focal length.

The design of the flexible zoom compound eye incorporates a regional zoom approach. However, traditional designs have limited flexibility and can only zoom within a certain range. To address this issue, J. J. Cao et al. developed a tunable bionic compound eye Cao et al. (2020). The structure is created using femtosecond laser wet etching technology. Initially, the silicon wafer template, which underwent laser processing, is etched in a solution containing hydrofluoric acid (HF), nitric acid (HNO_3_), and acetic acid (CH_3_COOH). Subsequently, the microstructure of the template is transferred onto a PDMS substrate through the demolding process. The resulting PDMS microlens array (MLA) is flexible and deformable, and it is integrated with a microfluidic chamber to enable zoom imaging, as shown in Figure 8A. By adjusting the volume of water injected into the microfluidic chamber, the compound eye can transition from a planar structure to a hemispherical shape, achieving a field of view (FOV) of 180° and a variable focal length ranging from 3.03 mm to infinity. The angular resolution of the compound eye is 3.86 × 10^−4^ rad, granting it the capability to detect targets at different distances, as depicted in Figures 8B–G.

**FIGURE 9:**
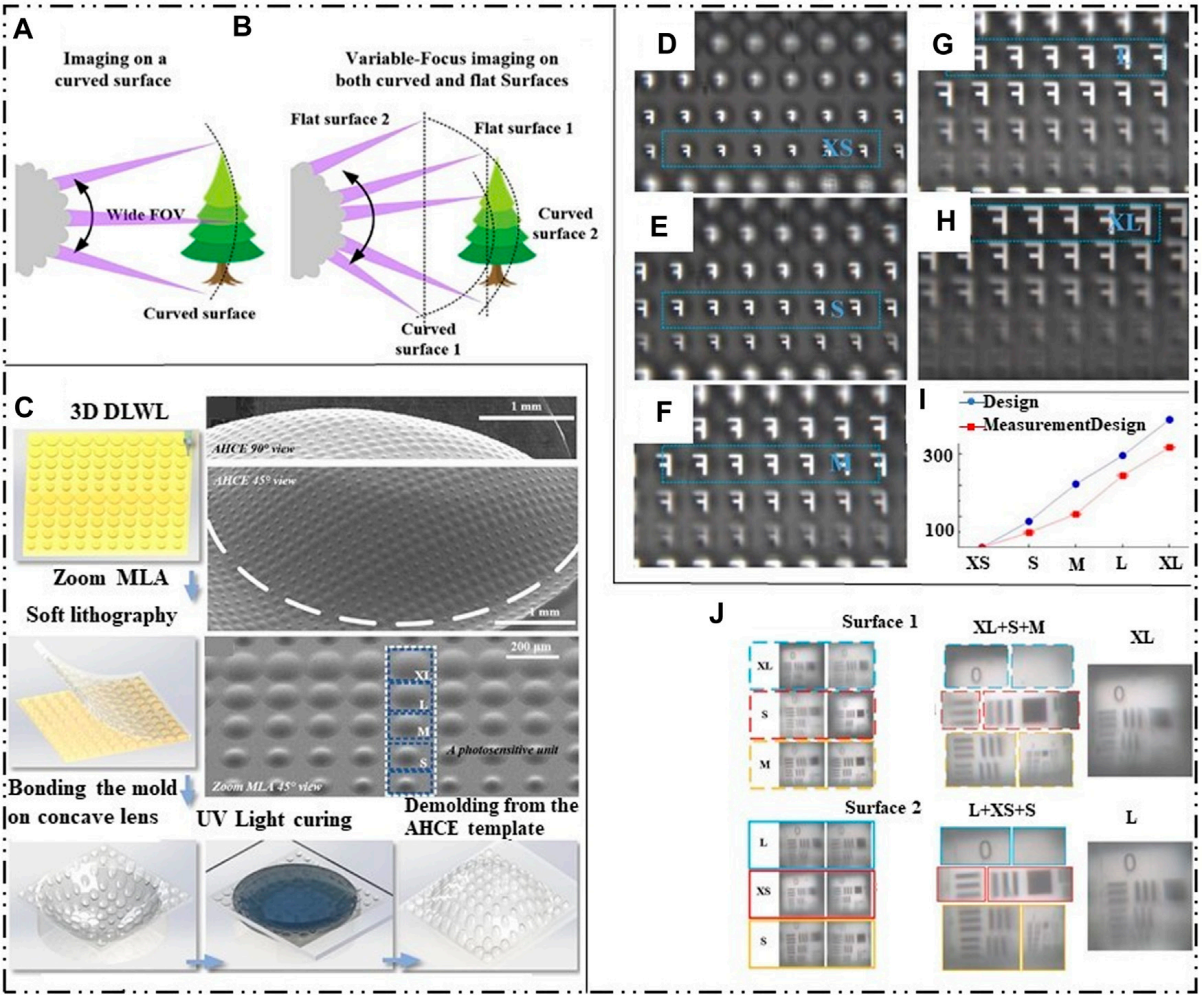
Femtosecond laser etching manufacturing zoom compound eye and inkjet printing and air assisted deformation manufacturing zoom compound eye diagram. [Reproduced with permission from (Luan et al., 2022)]. **(A)** AHCE curved surface zoom. **(B)** AHCE plane zoom image. **(C)** AHCE processing flow chart. **(D–H)** Curved zoom imaging at different distances. **(I)** Relative distance between different types of ommatidia and AHCE. **(J)** Planar compound eye imaging in different regions.

Although the tunable bionic compound eye can achieve flexible focal length, it needs to zoom through the solution volume, and the response time is slow, which limits the practical application of the compound eye. In response to this problem, S.Y. Luan et al. inspired by the bionic design of ancient trilobites, created an artificial super compound eye (AHCE) Luan et al. (2022). It is a microlens with five different curvatures, which can be regarded as a small eye with five different focal lengths. The surface and plane zoom functions are realized under the composition of five small eyes, as shown in Figures 9A, B. The structure obtains the planar zoom MLA of the required mold by laser direct writing technology and controlling the exposure position and intensity through the optical system. After putting the PDMS-coated zoom MLA into the oven, the soft film of the zoom MLA was obtained by demoulding or soft lithography. The soft film of zoom MLA is bonded to the concave lens to obtain the template of AHCE. Then, the cyclic olefin copolymer (COC) was poured on the PDMS on the concave lens and covered with a glass substrate. Finally, through UV curing and demolding, the artificial super compound eye lens is obtained as shown in Figure 9C. Since AHCE is composed of five small eyes with different focal lengths, the structure can not only perform zoom imaging on the surface, but also form zoom imaging functions on planes with different distances through information sharing and scaling functions, as shown in Figures 9D–J. J. Li et al. also proposed a curved compound eye lens based on inkjet printing and air-assisted deformation for the zoom response of the compound eye Li et al. (2023).

The development of curved zoom compound eye lenses has addressed the zoom problem encountered in artificial compound eyes and has played a significant role in integrating curved compound eye systems. However, the compound eye system often operates in humid or harsh environments, making it susceptible to a decline in imaging quality caused by water mist. Therefore, it is crucial to enhance the lens’s hydrophobicity and self-cleaning ability.

**FIGURE 10:**
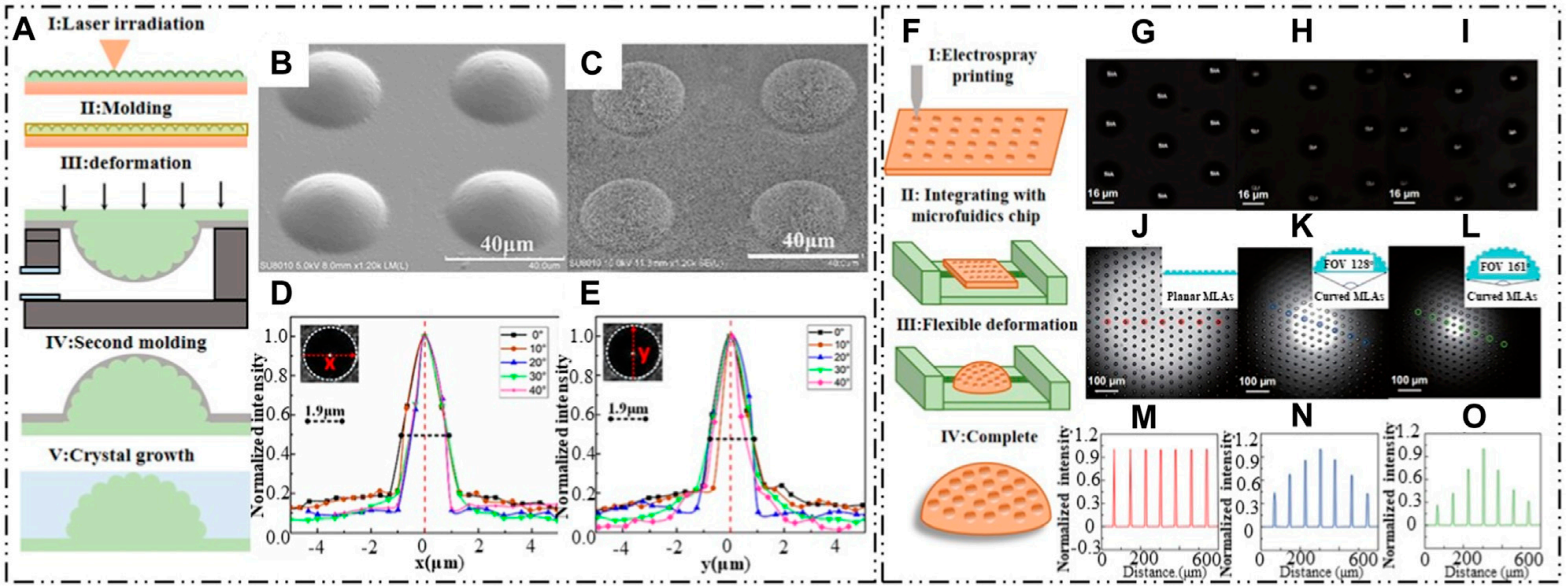
Laser swelling air assisted deformation technology and Jet printing with electrohy-drodynamic. (Reproduced with permission from (Li et al., 2019; Zhou et al., 2020)]. **(A)** Schematic of the fabrication process. **(B)** SEM image of convex structure after swelling. **(C)** SEM of fabricated bioinspired compound eye. **(D, E)** Intensity distribution of bioinspired compound eye along *x* and *y* direction, respectively. **(F)** Schematic of the fabrication process. **(G**–**I)** Imaging of “SIA” from center, middle and edge region. **(J–L)** Focusing ability text for tunable artificial compound eye with variable FOVs of 0°, 128° and 161°. **(M–O)** Intensity distribution of the focal spots from the ommatidia in regions corresponding to **(J–L)**.

In a study by J. Li et al., a combination of modified laser swelling, air-assisted techniques, and controlled crystal growth was employed to create an artificial compound eye without distortion Li et al. (2019). Initially, a microlens array was fabricated using laser swelling technology on a substrate. The convex array template was then fixed onto 184 elastic slabs. By attaching a concave membrane to a circular chamber connected to a vacuum pump, the membrane was deformed from a plane to a surface. The process is shown in Figure 10A. This process resulted in a bioinspired compound eye with nano structures, as observed in the scanning electron microscope (SEM) image shown in Figures 10B, C. The introduced nano structures effectively reduced surface reflectance and improved surface hydrophobicity. The bioinspired compound eye exhibited a nearly normal incidence distribution along the *x* and *y* directions, with a decrease in central light intensity as the angle of incidence increased. It demonstrated excellent imaging properties and focusing ability under tilted incident light.

Another approach using an electro-hydrodynamic jet printing technology for manufacturing waterproof artificial compound eyes was proposed by Zhou et al. (2020). The processing scheme, as illustrated in Figure 10F, involved spraying a microlens array onto a flexible PDMS film and deforming the film using an integrated microfluidics chip to create the artificial compound eye with a variable field of view (FOV). The flexible film, consisting of a hybrid of microlens and nanolens arrays, exhibited superb superhydrophobic properties with a water contact angle of 158°. Experimental tests on the imaging ability were conducted, and Figures 10G–I shows the imaging results of the letters “SIA” captured from the center, middle, and edge regions. The focused lens at the center of the microlens array can be clearly observed, as the film transitions from a planar to a curved surface upon deformation. Figures 10J–L depicts the deformation state of the microlens array film for the artificial compound eyes, and Figures 10M–O illustrates the intensity distribution of the circled positions. The microfluidics chip enabled the creation of artificial compound eyes with a variable FOV ranging from 0° to 160°.

**FIGURE 11:**
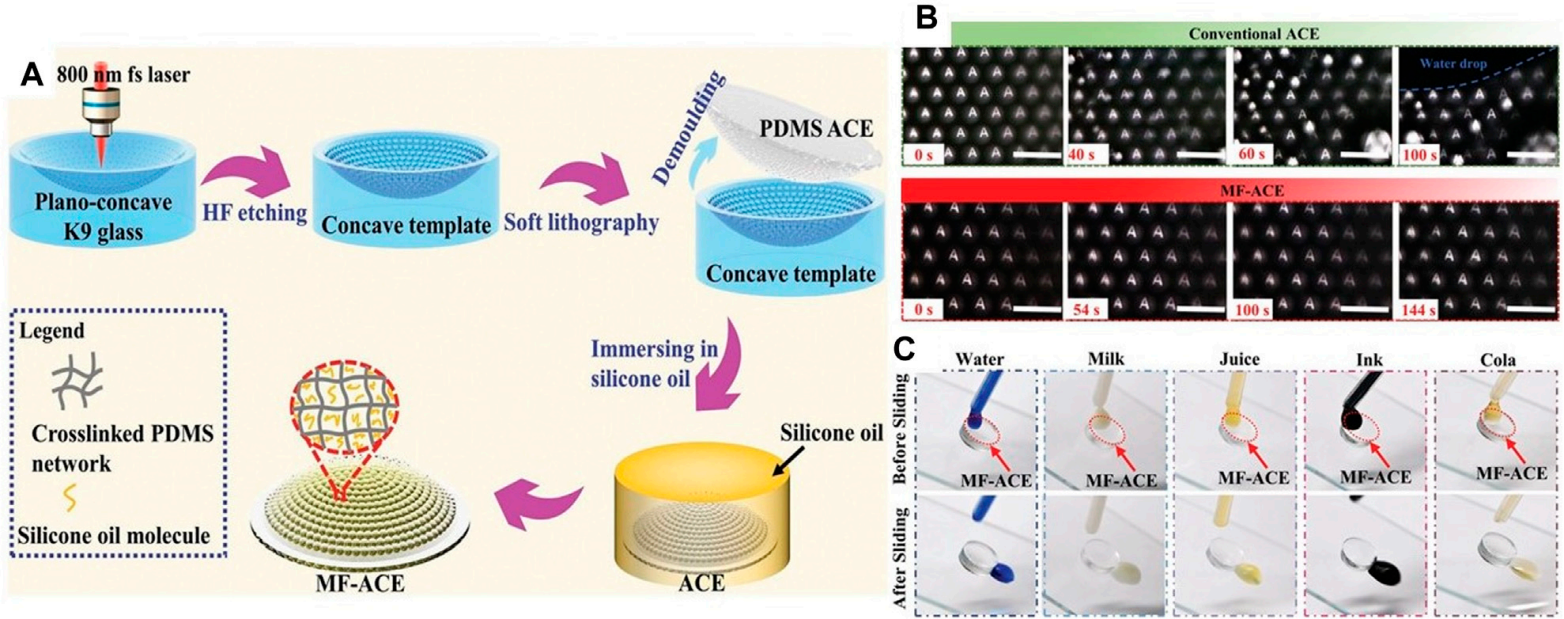
The schematic diagram of anti-fog and antifouling compound eye made by femtosecond laser wet etching method. [Reproduced with permission from (Li et al., 2022)]. **(A)** Anti-fouling and anti-fog compound eye processing flow chart. **(B)** 0–144 s continuous water mist exposure imaging of compound eye lens. **(C)** Antifouling ability test of compound eye under different solutions.

Although the above two kinds of compound eyes can achieve hydrophobicity, the superhydrophobic surface is obtained at the expense of the transparency of the compound eye, and the micro-nano manufacturing process used is not conducive to large-scale production. At the same time, when subjected to high pressure, long-term work or wear, these superwetting surfaces have poor stability, which can lead to the loss of hydrophobicity. In response to this problem, M.J. Li et al. inspired by the Nepenthes capture, designed an anti-fog and anti-fouling compound eye structure Li et al. (2022). The structure uses femtosecond laser wet etching method to, etch the required microstructure template on the curved K9 optical glass, apply PDMS to the template, and obtain the required ACE after cooling and demoulding. The structure uses femtosecond laser wet etching method to, etch the required microstructure template on the curved K9 optical glass, apply PDMS to the template, and obtain the required ACE after cooling and demoulding. ACE was immersed in silicone oil for several hours to form a smooth surface injected with lubricant. The processing flow is shown in Figure 11A. The compound eye structure integrates more than 3,000 microlenses, which not only achieves 64°FOV, but also has a spatial resolution of 50.8 lp mm^–1^. It can withstand continuous water mist exposure of 1.45 mL min^–1^ at least 144s as shown in Figure 11B. The structure has high self-cleaning performance and can repel complex liquids as shown in Figure 11C. It has great potential in autonomous driving, medical or visual systems in harsh environments.

The compound visual organs mentioned earlier are fabricated mainly from synthetic polymers. But regretfully, this restricts their potential applicability due to their susceptibility to distortion when exposed to elevated temperatures and intense pressures. Conversely, artificial compound eyes constructed from glass present a promising prospect for expansive imaging and swift detection, owing to their extraordinary thermal and mechanical resiliency.

**FIGURE 12:**
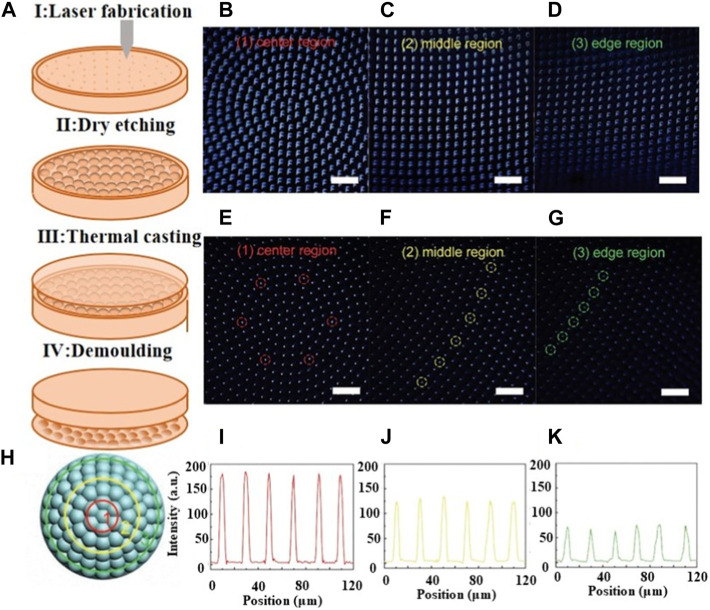
Femtosecond laser direct writing with dry etching assisted [Reproduced with permission from (Liu et al., 2019)]. **(A)** Schematic of the fabrication process. **(B**–**D)** Imaging of “F” from three regions. **(E**–**G)** Focusing properties from three regions. **(H)** The schematic diagram of the measured regions. **(I**–**K)** Intensities of the focal spots from the ommatidia in regions corresponding to **(E**–**G)**.

To address this challenge, X.Q. Liu et al. proposed a technology that utilizes sapphire concave compound eyes as high-temperature and hard-casting templates Liu et al. (2019a). A dry etching assisted femtosecond laser processing method was used to create centimeter-sized concave compound eyes on a curved sapphire substrate. The process involved carving microholes on the curved sapphire substrate through femtosecond laser scanning, followed by dry etching to form the concave compound eyes. By employing a femtosecond laser manufacturing system, they rapidly fabricated a uniform concave compound eye with a diameter of 20 μm and a depth of 1.5 μm. Subsequently, a glass compound eye was created using a high-temperature casting method, as illustrated in Figure 12A. The resulting compound eye produced clear images of the letter “F” from the center, middle, and edge regions, as shown in Figures 12B–D. When the objective lens was adjusted from far to near, clear images of the letter “F” could be observed across the compound eye, from the center to the edge. Similarly, the focus points were clear in all three regions when the objective lens was adjusted from far to near. Figures 12I–K demonstrates the corresponding intensities, with the grayscale intensities of the focal spots being nearly identical on the same focal plane formed by ommatidia at the same distance from the center. Compared to direct laser ablation, this approach significantly improves processing efficiency by more than two orders of magnitude. The combination of dry etching assisted femtosecond laser machining and casting replication technologies holds promising prospects for micro/nanofabrication of hard materials.

Table 2 presents a comparison of microfabrication technologies for miniature curved compound eyes. The technologies were evaluated based on processing efficiency, surface quality, 3D fabrication capacity, and the reported *d*_*o*_. Among these technologies femtosecond laser direct writing involves scanning the contour point by point, resulting in lower processing efficiency. However, it offers higher 3D flexibility compared to other methods. Surface quality is primarily influenced by the control of stair effects and the self-smoothing effect of materials. Ultra-precision machining, as a true three-dimensional processing method, holds a significant position in microlens manufacturing. Nevertheless, the wear of the tool over time can reduce machining accuracy and increase processing costs. Femtosecond laser wet etching with thermal embossing, modified laser swelling with air-assisted techniques, and inkjet printing with air-assisted microfabrication are all based on bending the flexible planar compound eye using physical force. The mold reproduction method enhances machining efficiency for planar compound eyes, but it limits the contour of curved compound eyes to simple curved surfaces due to the expansion caused by physical forces. Jet printing with electrohydrodynamics is a non-contact printing technology that employs an electric field to trigger ink injection onto the substrate. Surface quality mainly depends on material shrinkage before and after curing. Femtosecond laser-enhanced wet and dry etching can produce fine molds with a certain level of 3D complexity. Surface quality relies primarily on the mold’s surface quality and the material surface’s adhesion.

**TABLE 2:** Microfabrication technologies for miniature curved compound eye.

Fabrication technology		Processing efficiency	Surface quality	3D capacity	Reported *d*_*o*_
Femtosecond laser two-photon polymerization	BSA Smart Compound Eyes	Low	Medium	High	8 μm (Ma et al., 2019)
	Voxel-regulated compound eye				16 μm (Wu et al., 2014)
Molding method	Flexible zoom compound eye	High	Medium	Medium	∼14 μm (Li et al., 2018)
Femtosecond laser-enhanced wet etching	Tunable bionic compound eye	Medium	Medium	Medium	50 μm (Cao et al., 2020)
	Anti-fog anti-fouling compound eye				108 μm (Li et al., 2022)
	3D glass compound eye				20 μm (Liu et al., 2019a)
	Artificial super-compound eye lens				80–160 μm (Luan et al., 2022)
Inkjet printing with air-assisted deformation method	Inkjet printing with air-assisted deformation compound eye	Medium	Medium	Low	25–75 μm (Li et al., 2023)
Modified laser swelling with air-assisted	Air-assisted modified laser swelling compound eye	High	Medium	Low	40 μm (Li et al., 2019)
Jet printing with electro-hydrodynamic	Electric inkjet printing compound eye	Medium	High	Low	∼8 μm (Zhou et al., 2020)

## 4 Photoelectric detection of miniature compound eye

To achieve high-precision detection in miniature compound eye imaging, it is crucial to have precise matching between the compound eye and the photoelectric sensor. Currently, commercially available photoelectric sensors are planar, while advanced miniature compound eyes have curved surfaces. This creates a mismatch between the focal plane of the compound eye and the planar photoelectric sensor. To address this issue, there are two main approaches. The first approach is to develop miniature curved photoelectric sensors. This involves designing and manufacturing sensors with a curved surface that matches the curvature of the compound eye. By doing so, the focal plane of the sensor can align with the compound eye, improving the overall imaging precision. The second approach is to modify the structure of the compound eye itself. Researchers can explore techniques to adjust the focal plane of the compound eye to match the planar photoelectric sensor. This may involve manipulating the shape or arrangement of the individual optical elements within the compound eye or incorporating additional optical components to redirect the light onto the planar sensor. Both approaches aim to achieve a better alignment between the compound eye and the photoelectric sensor, improving the precision of detection in miniature compound eye imaging.

### 4.1 Microfabrication technologies for planar photoelectric detection

A series of solutions have been proposed for planar photoelectric detection, for example, focal distance-tunable flexible materials (Ma et al., 2019), self-writing waveguide (Kim et al., 2005), optical fiber guidance (Liu et al., 2019), refractive lens array (Li and Yi, 2012; Jing et al., 2021), multiple spherical microlenses array (Jing et al., 2022) and so on.

**FIGURE 13:**
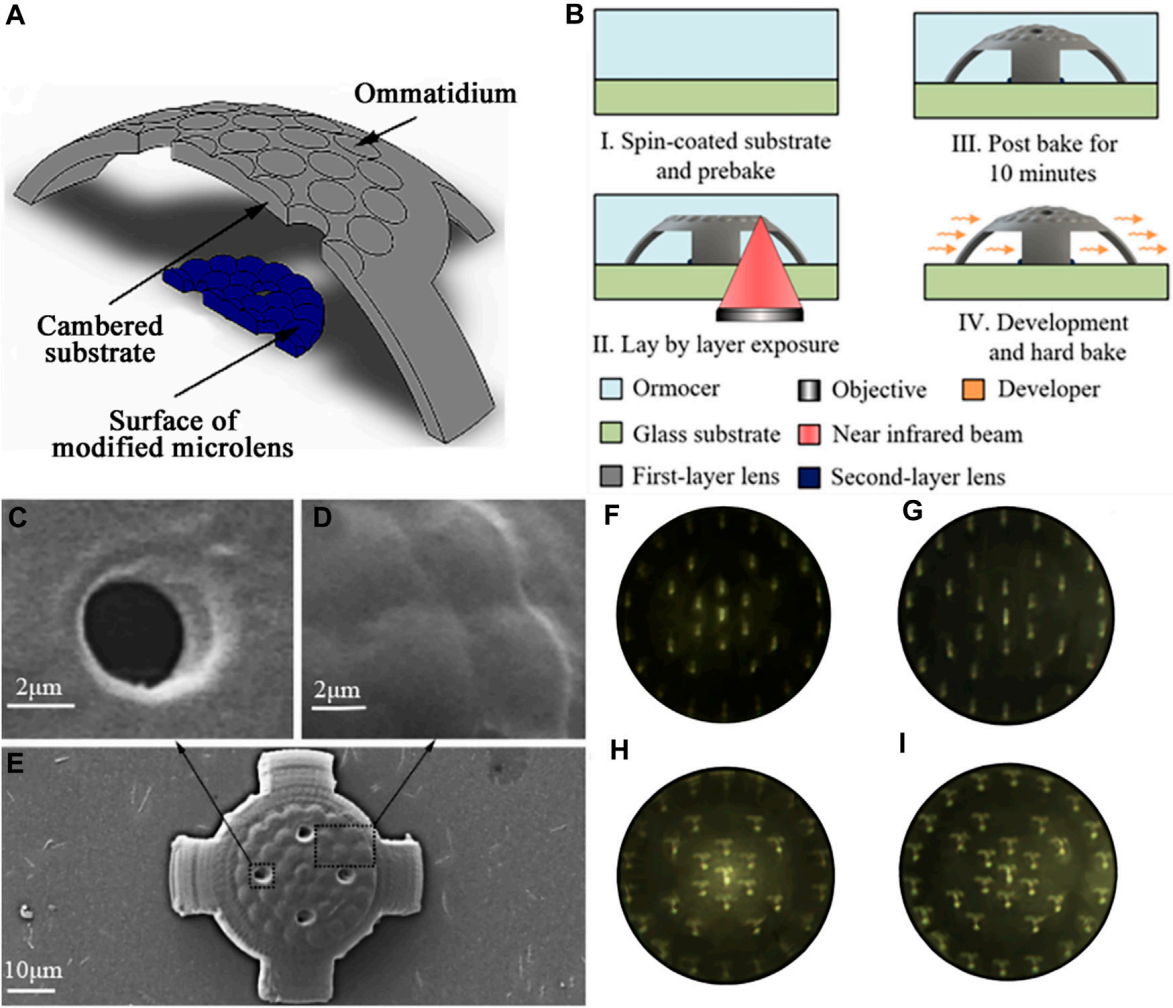
The schematic diagram of processing and imaging [Reproduced with permission from (Jing et al., 2021)]. **(A)** The structure of the DLCCE. **(B)** Schematic of the fabrication process. **(C)** Partial enlarged detail of through-hole. **(D)** Partial enlarged detail of ommatidia. **(E)** SEM image of the compound eye. **(F)** Detected “I” image from SLCCE. **(G)** Detected “I” image from DLCCE. **(H)** Detected “T” image from SLCCE. **(I)** Detected “T” image from DLCCE.

A double-layer curved compound eye (DLCCE) was proposed by (Jing et al., 2022) to solve the existing problems. For each ommatidium, a corresponding modified microlens was designed to refract and focus the incident rays on the photoelectric detector. The structure of the double-layer curved compound eye is shown in Figure 13A. The DLCCE was fabricated by two-photon polymerization microfabrication technology. The fabrication processing is shown in Figure 13B. Figures 13C, D show the partial enlarged detail of a through-hole and ommatidia, respectively. The imaging comparisons of single-layer curved compound eye (SLCCE) and DLCCE are shown in Figures 13F–I. Figures 13F, H show the imaging of “I” and “T” of SLCCE, respectively, the imaging of “I” and “T” of DLCCE are shown in Figures 13G, I. The DLCCE shows a uniform light intensity at each level (Jing et al., 2021). Although two-photon polymerization microfabrication can achieve the integrated fabrication of double-layer microlens directly, the imaging quality also suffers from processing errors of two layers.

**FIGURE 14:**
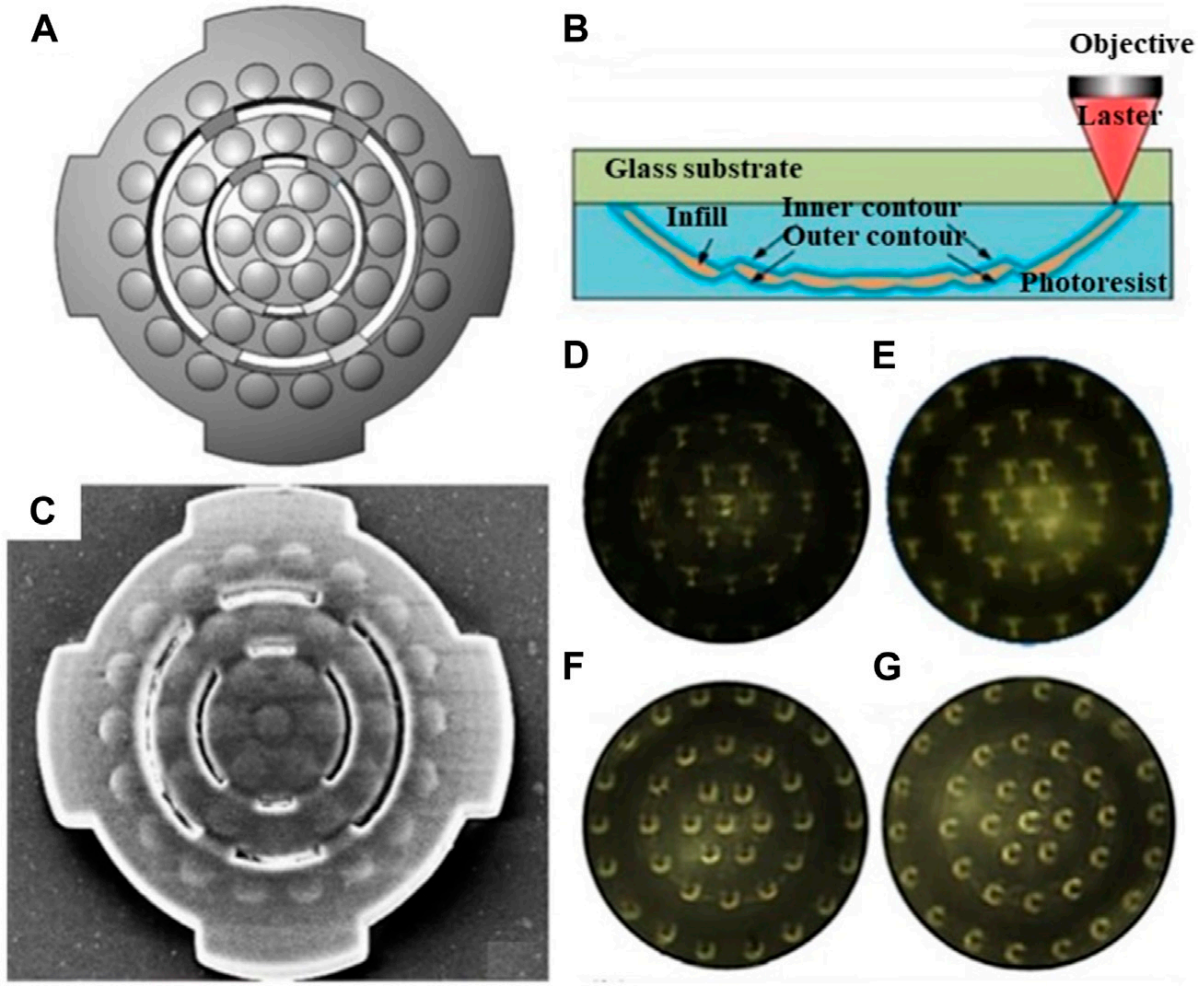
The structure and imaging detection of multi-spherical compound eye [Reproduced with permission from (Jing et al., 2022)]. **(A)** The structure chart of the multi-spherical compound eye. **(B)** Schematic of the fabrication process. **(C)** SEM image of the multi-spherical compound eye. **(D)** Imaging of “T” through single-spherical compound eye. **(E)** Imaging of “T” through single-spherical compound eye. **(F)** Imaging of “U” through single-spherical compound eye. **(G)** Imaging of “C” through single-spherical compound eye.

Furthermore, an ultra-compact micro multi-spherical compound eye (MSCE) was proposed to achieve the focus on the planar photoelectric detector. The structure of the MSCE is shown in Figure 14A. Through adjusting the structural distance along the optical axis of each ommatidium at each level, it can achieve the synchronous focus of all the ommatidia on the planar photoelectric detector with a single-layer compound eye, but without additional adjustable components. The MSCE was fabricated by two-photon polymerization technology. The fabrication processing is shown in Figure 14B. SEM image of the compound eye is shown in Figure 14C. The image of single-spherical compound eye (SSCE) in Figure 14D was used to compare with MSCE. Figures 14E–G show the imaging of “T,” “U,” “C” detected by MSCE. The results show relatively good imaging quality at the edge of MSCE, and the intensity is uniform at each level (Jing et al., 2022). The MSCE structure requires a high 3D controllability of microfabrication technology.

**FIGURE 15:**
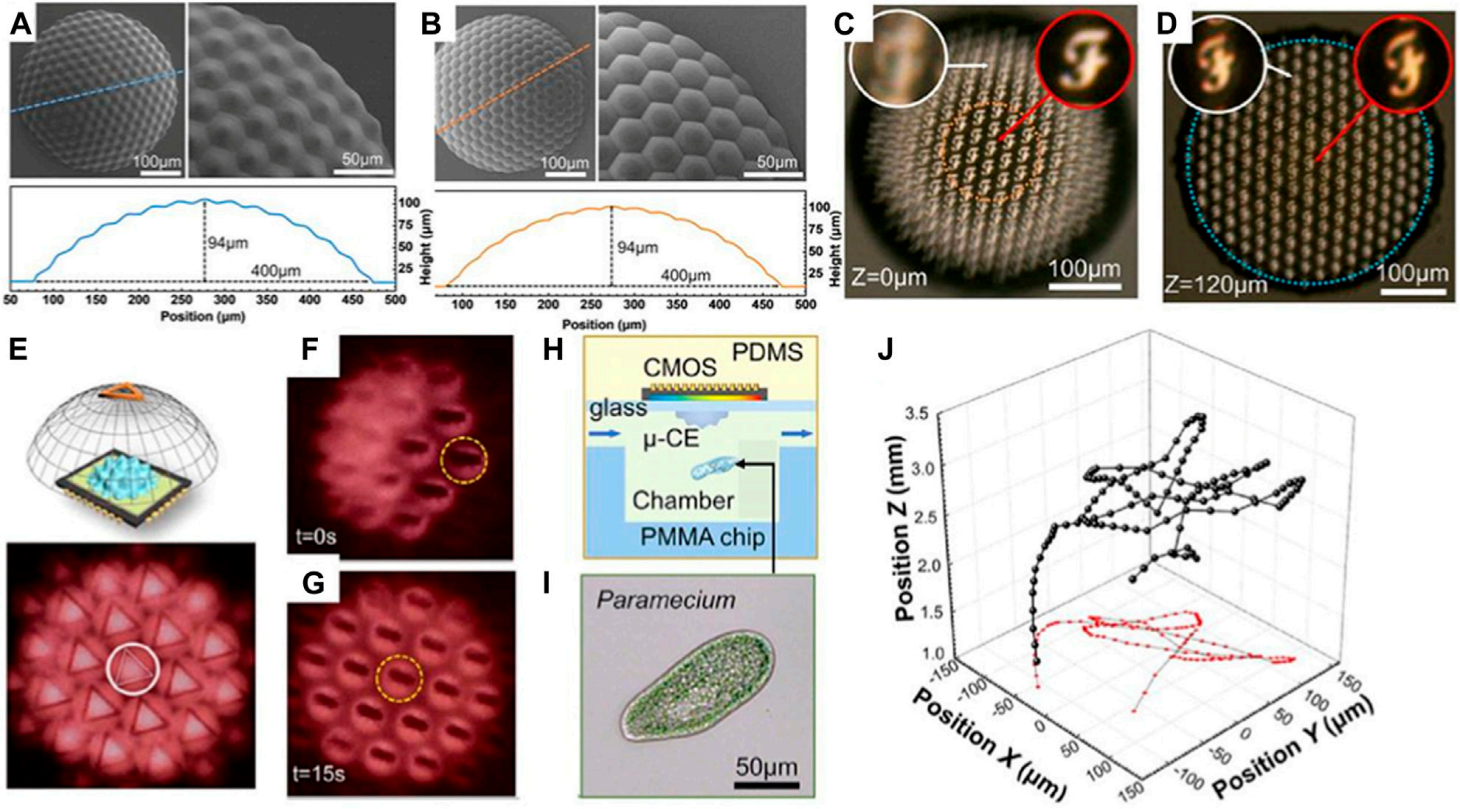
Micro photoelectric compound eye camera. (Reproduced with permission from (Hu et al., 2022)). **(A)** Logarithmic CEs. **(B)** Spherical CEs. **(C)** Spherical compound eye imaging. **(D)** Logarithmic compound eye imaging. **(E)** μ-CE space recognition. **(F)** Beetle motion map of μ-CE at 0s. **(G)**Beetle movement diagram at 15s. **(H)** μ-CE microbial detection diagram. **(I)** Microscopic images of a *paramecium*. **(J)** The 3D trajectory of the *paramecium*.

Hu et al. (2022) also proposed a miniature photoelectric compound eye camera (μ-CE) for planar sensors (Hu et al., 2022). The camera utilizes a uniformly logarithmic profile, as depicted in Figure 15A, eliminating the need to calculate the focal lengths of individual small eyes. This facilitates the design and manufacturing process, even with an increased number of compound eyes. Unlike spherical compound eyes, shown in Figure 15B, the logarithmic configuration of μ-CE extends the focusing range, albeit resulting in slight dimming of the image. Nonetheless, this unique focusing characteristic effectively eliminates defocus issues, as shown in Figures 15C, D. The μ-CE has a compact structural size of approximately 400 μm, similar to that of a mosquito’s compound eye, and weighs only 230 mg. Additionally, it enables wide-angle imaging up to 90°, which is crucial for object recognition and trajectory detection in space, as demonstrated in Figures 15E–G. Due to its small size and excellent imaging capabilities, the μ-CE can be seamlessly integrated with microfluidic devices for live microorganism detection, as displayed in Figures 15H–J. Furthermore, M.C. Ma et al. designed a single-pixel superimposed compound eye system (SPSCE) with integrated microlenses, optical fibres and photoreceptors to reduce the complexity of optical design and develop an artificial superimposed compound eye system with higher imaging resolution Ma et al. (2021).

### 4.2 Microfabrication technologies for curved photoelectric detection

Besides adjusting the microlens array, the development of curved photoelectric detectors is another effective approach to enable simultaneous imaging of all the ommatidia within the curved compound eye.

**FIGURE 16:**
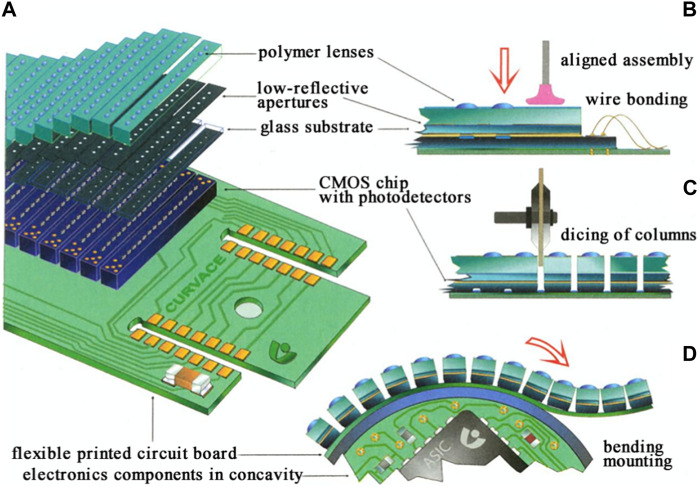
Design and assembly of curved compound eye [Reproduced with permission from (Floreano et al., 2013)]. **(A)** Assembly schematic of the curved compound eye. **(B)** Accurate alignment and assembly process. **(C)** Dicing of the assembled array in columns down to the flexible interconnection layer, which remains intact. **(D)** Curving of the ommatidial array.

D. Floreano et al. presented a miniature curved artificial compound eye with flexible printed circuit board, the layers of the curved compound eye are shown in Figure 16A Floreano et al. (2013). The design consists of three layers of arrays: a microlens array, a photoelectric detector array and a curved flexible imager. Figure 16B illustrates the precise alignment and assembly process. Figure 16C demonstrates the dicing of the assembled array into columns, down to the flexible interconnection layer, which remains undamaged. Figure 16D depicts the curvature of the ommatidial array. The designed artificial compound eye bearing a hemispherical field of view with embedded and programmable low-power signal processing, high temporal resolution, and local adaptation to illumination. Y.M. Song et al. presented a camera design inspired by arthropods, featuring nearly full hemispherical shapes Song et al. (2013). The surface of the digital camera is densely populated with imaging elements, with 180 artificial ommatidia, comparable to the eyes of fire ants and bark beetles.

## 5 Application and outlook

### 5.1 Applications of artificial compound eyes

The purpose of bionic compound eyes is to replicate the structures, characteristics, and behaviors of natural compound eyes, with the aim of providing valuable insights for designing innovative optical systems applicable to various facets of manufacturing and daily existence. The utilization of artificial compound eyes has found widespread application across diverse domains, owing to their advantageous attributes such as an expansive field of view, heightened sensitivity, boundless depth of field, and other remarkable features. These domains encompass large FOV perception (Huang et al., 2014; Cogal and Leblebici, 2017; Wang et al., 2019), fast recognition (Shi et al., 2017; Xu et al., 2020), high depth of field (DOF) perception (Kagawa et al., 2012a; Lee and Lee, 2018), spectral perception (Rui et al., 2004; Jin et al., 2013; Chen et al., 2017; Cao et al., 2019; Yu et al., 2020; Zhang et al., 2021), polarization imaging (Kagawa et al., 2012b; Kagawa et al., 2012c; Miyata et al., 2020), and numerous others.

#### 5.1.1 Large FOV perception

**FIGURE 17:**
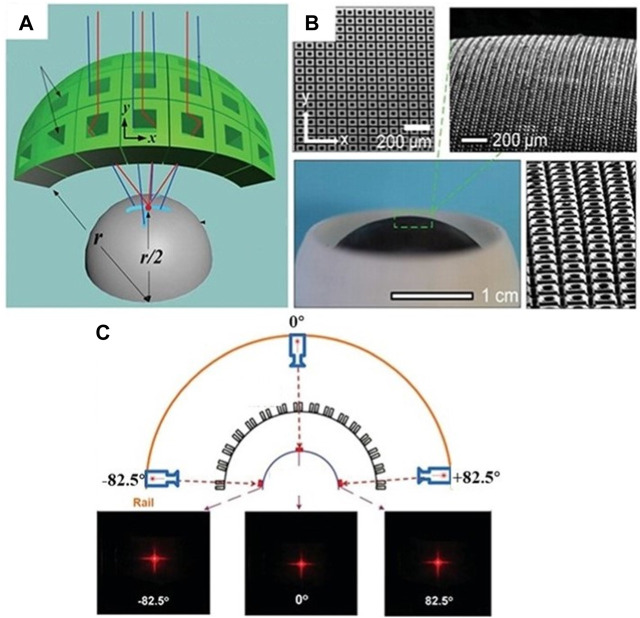
Large FOV perception. (Reproduced with permission from (Huang et al., 2014)). **(A)** Schematic diagram of RSCE imaging. **(B)** Images of the detailed microstructures of a 3D hemispherical artificial RSCE. **(C)** Imaging performance of RSCE with a FOV of 165°.

Artificial compound eyes can achieve a large FOV perception, similar to the compound eyes of insects in the natural world, through the arrangement of micro lenses on a curved substrate. Indeed, this is also the main reason why curved compound eyes have received more attention in recent years compared to planar compound eyes. C.C. Huang et al. proposed reflect superposition compound eyes (RSCEs), which exhibit minimal chromatic aberration, an exceptional field of view of up to 165° without distortion, moderate aberrations, and comparable imaging quality Huang et al. (2014). Figure 17A illustrates the schematic diagram of RSCE imaging. The detailed structure of the designed RSCE is shown in Figure 17B. Figure 17C showcases the imaging performance of RSCE with a 165° FOV. O. Cogal et al. proposed a multi-camera endoscopic compound eye with a 180° FOV and a radius size of 18 mm Cogal and Leblebici (2017). This system can capture 25 frames per second (fps) video with a resolution of 1,080 × 1,080 pixels, operating at a processing clock frequency of 120 MHz.

#### 5.1.2 Fast recognition

**FIGURE 18:**
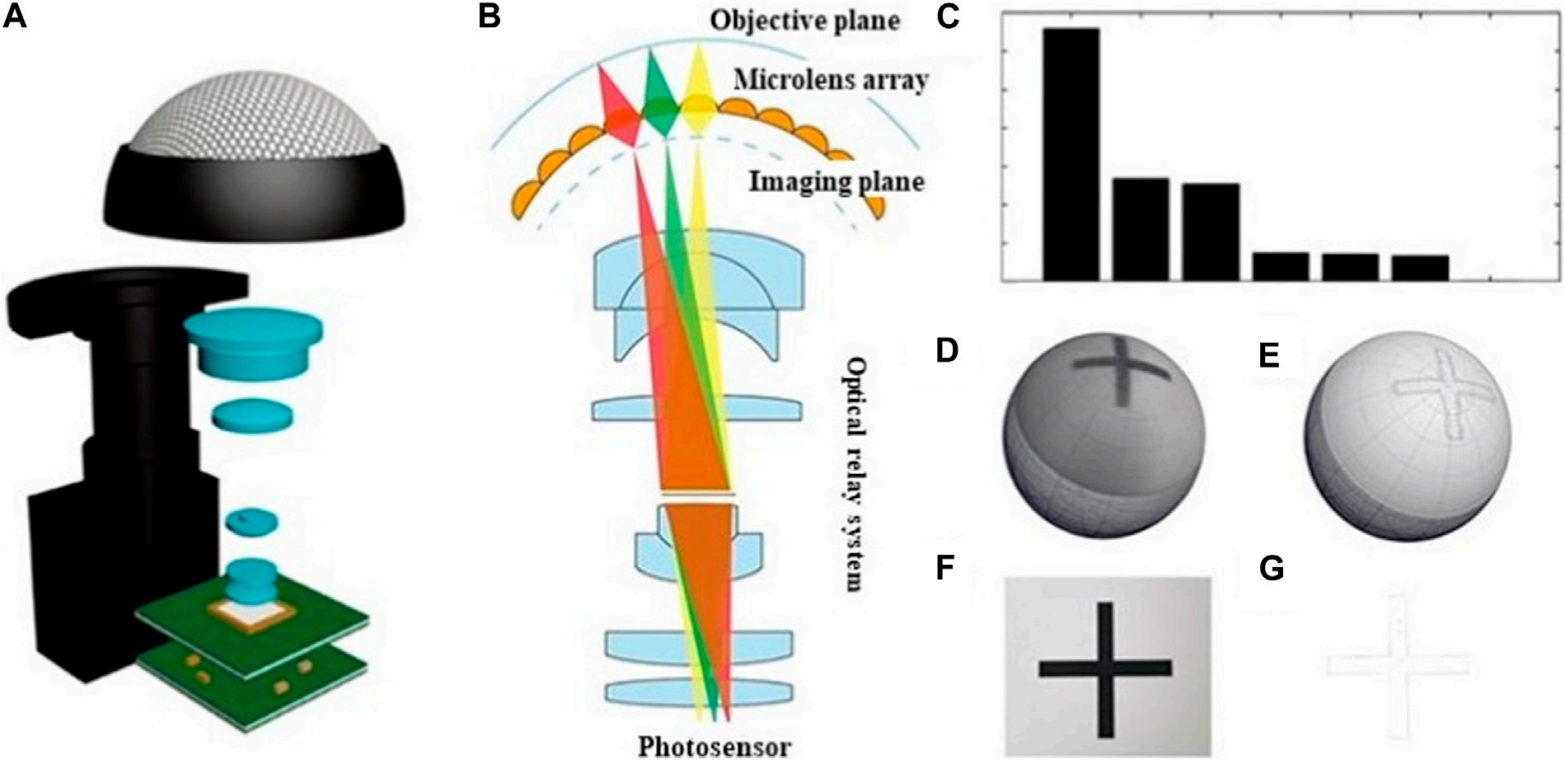
Fast detection and capture of object. [Reproduced with permission from (Shi et al., 2017)]. **(A)** Exploded view of the SCECam. **(B)** Schematic illustration of SCECam imaging priciple. **(C)** Processing speed comparison of different methods. **(D)** Raw image of symbol “+” captured by SCECam. **(E)** The image obtained by edge detection based on SCECam method. **(F)** Raw image of symbol “+” captured by one conventional camera with same resolution as the SCECam. **(G)** The image obtained by the Canny edge-detection method.

Compared to single-chambered eyes, compound eyes have a tendency to perceive shapes and outlines rather than clear and precise details. However, compound eyes have the advantage of fast recognition imaging due to the overlapping FOV between adjacent ommatidia. C.Y. Shi et al. proposed a kind of hemispherical compound eye camera (SCECam), which utilizes the imaging differences of neighboring ommatidia to recognize objects Shi et al. (2017). Figures 18A, B shows the exploded view and imaging principle of SCECam. The recognition speed of SCECam is significantly faster compared to conventional methods such as Canny and Log edge-detection techniques, with an improvement of two to three orders of magnitude. The running time of Canny, Zerocross, Log, Sobel, Prewitt Robert and SCECam edge detection method is shown in Figure 18C. Figures 18D–G display the edge detection results for SCECam method and conventional Canny edge detection method, respectively. Another advancement in this field is the design and fabrication of a biomimetic curved compound-eye camera (BCCEC) by Xu et al. (2020). This camera successfully detected and captured a moving silver box measuring 230 mm × 250 mm at a distance of 25 m. The algorithm used in this camera was optimized based on the fast discriminative scale space tracker, resulting in improved detection and capture speed.

#### 5.1.3 High DOF perception

**FIGURE 19:**
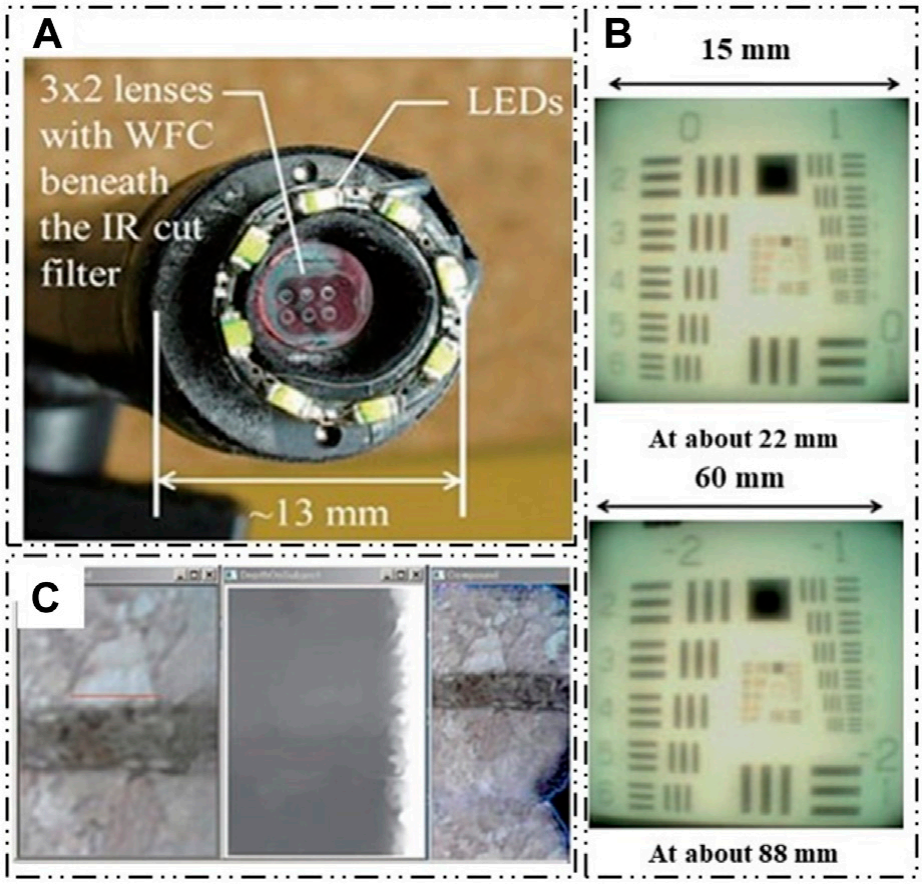
High DOF perception. [Reproduced with permission from (Kagawa et al., 2012a)]. **(A)** TOMBO compound-eye camera. **(B)** The images of 1951 USAF test chart detected by TOMBO camera. **(C)** The all-focus image and the estimated depth map of the cork board sample.

The depth cues can be derived from the subtle variations in the views of adjacent ommatidia in a compound eye, especially when the ommatidia are closely positioned and have overlapping fields of view. K. Kagawa et al. proposed a compound-eye camera TOMBO (Thin Observation Module by Bound Optics) inspired by the insect eye structure as an endoscope capable of acquiring depth information Kagawa et al. (2012a). Figure 19A shows the TOMBO endoscope prototype. A wavefront coding (WFC) technique and a 640 × 480-pixel color imager was used to demonstrate extended depth of field. Figure 19B shows the images of 1951 USAF test chart detected by TOMBO camera. The results show that almost constant resolution is provided at distances to the object of 20–80 mm. Figure 19C show the all-focus image and the estimated depth map of the cork board sample. W.B. Lee et al. proposed a method for estimating object depths in a monocular compound eye imaging system based on the computational compound eye (COMPU-EYE) framework Lee and Lee (2018). In the COMPU-EYE system, acceptance angles are considerably larger than interommatidial angles, causing overlap between the ommatidial receptive fields. In the proposed depth estimation technique, the disparities between these receptive fields are used to determine object distances.

#### 5.1.4 Spectral perception

Multispectral perception technology can achieve accurate spectral separation and obtain different band image information. Combined with digital image processing technology, it can reveal the hidden information that is difficult to observe by ordinary cameras and accurately reproduce the color, luster, and texture of the real-world objects.

**FIGURE 20:**
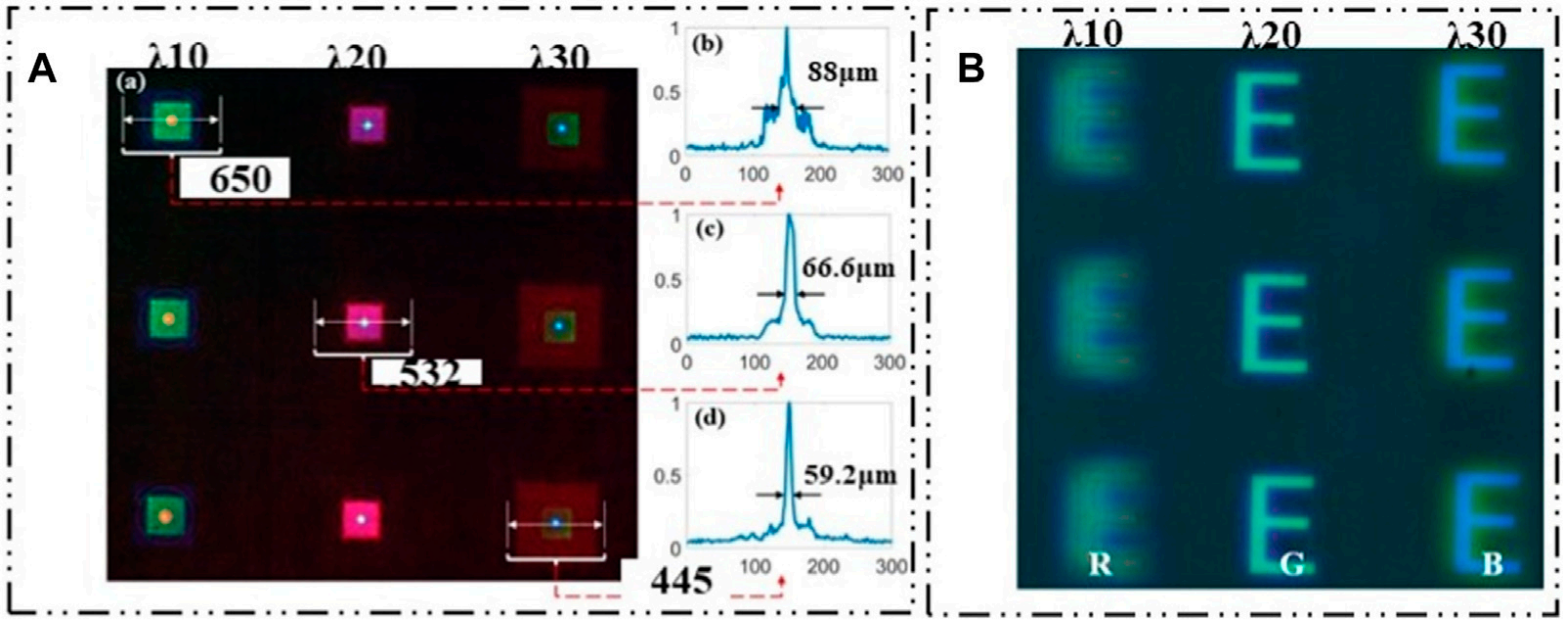
Planar spectral imaging. [Reproduced with permission from (Cao et al., 2019)]. **(A)** Distribution of red, green and blue focus. **(B)** Red-green-blue multispectral imaging effect.

S.J. Rui et al. presented an optimized multispectral TOMBO camera capable of capturing spectral information in different spectral bands of a target by utilizing multiple photodetectors to observe the same point Rui et al. )(2004). J. Jian *et al.* proposed a new method for the integration of microlens array and multi-channel filter based on MEMS Jin et al. (2013). The structure has multiple optical units, each of which can capture the information of a certain band. By combining with CMOS photodetectors, a multi-spectral imaging system is established. Due to the various production processes of multi-channel filters by pigment dispersion method, the large-scale production efficiency is low. Based on micro-electro-mechanical system (MEMS) technology, A.X. Cao et al. fabricated a compact structure that integrates a microlens array with a multi-channel filter, which can simultaneously achieve imaging and spectral functions through a diffractive beam splitter lens Cao et al. (2019). Diffractive spectral imaging is a staring imaging technology based on diffractive beam splitting lens, which images the scene in the field of view and reads it once through the focal plane array detector. As shown in Figures 20A, B. The spectral image received by the detector is the focused image of a single wavelength at the focal plane and the defocused image of other wavelengths at this position, which will cause interference and blur to the image. The image needs to be processed to obtain a clearer spectral image.

The field of view of the planar spectral compound eye is small, and the poor imaging quality of the lens edge seriously hinders its wide application. The curved ACE can effectively solve the problem.

**FIGURE 21:**
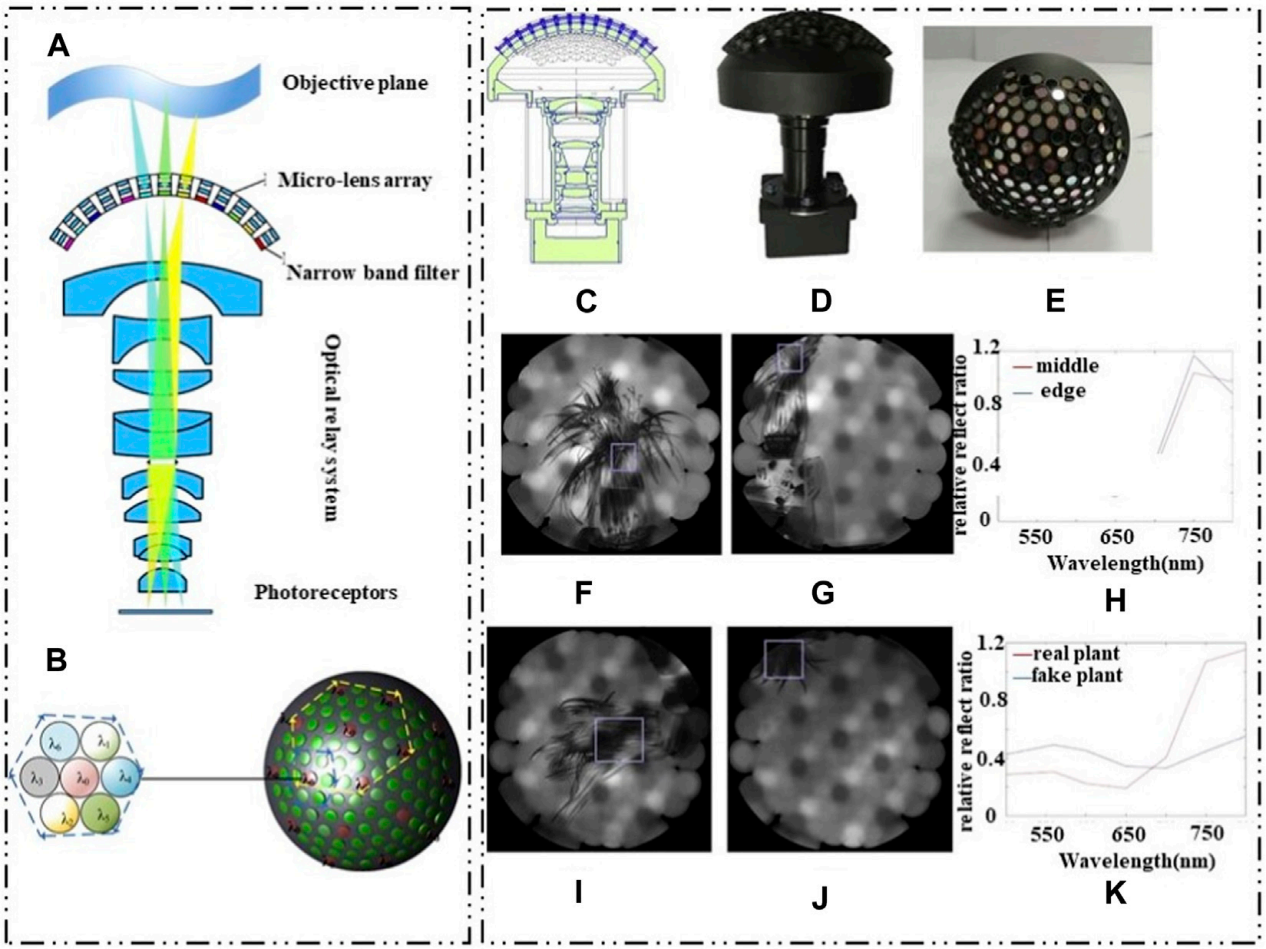
Curved spectral compound eye imaging. (Reproduced with permission from (Yu et al., 2020; Zhang et al., 2021)). **(A)** MCCEC cross section diagram. **(B)** MCCEC multi-spectral channel layout. **(C)** Bionic multispectral bending compound eye camera structure diagram. **(D)** Bionic multispectral curved compound eye camera assembly original image. **(E)** Multispectral curved compound eye display. **(F)** Spectral imaging of the intermediate field of view. **(G)** Spectral imaging of the edge field of view. **(H)** Spectral curves under different field of view. **(I)** Real plant spectra. **(J)** Pseudo-plant spectra. **(K)** Spectral reflectance of true and false plants.

J.W. Chen et al. used a hybrid imprinting method to fabricate a multilayer micro-nano optical structure on a non-planar substrate, that is, a multilayer artificial compound eye multispectral imaging system Chen et al. (2017b). The system can realize curved surface spectral imaging, but the system configuration and image processing complexity are high, and the field of view is relatively small. X.D. Yu et al. proposed a new multi-spectral curved compound eye camera (MCCEC) Yu et al. (2020). The system consists of a narrow-band filter, an optical transformation subsystem, and an image sensor. The system can achieve multi-spectral imaging in a large field of view and obtain information in multiple spectral bands. The system has a super large field of view of 120°, which can realize multi-spectral imaging of seven bands, and solve a series of problems such as small field of view. The optical design of the system is shown in Figures 21A, B. According to the new curved surface spectral imaging system proposed by X.D. Yu et al., Y.J. Zhang et al. made further innovations for this system, improved the focal length of the original system, so that it can meet the long-distance work, with high resolution Zhang et al. (2021). The curved image plane formed by the curved compound eye is converted into a plane image plane by using an optical relay system. The Dalsa C5180M industrial camera with a total pixel of 5,120 × 5,120 and a pixel size of 4.5 μm × 4.5 μm is used as the image sensor. The whole sample is shown in Figures 21C–E. The system maintains the advantages provided by the curved compound eye structure, such as large field of view and special multi-aperture imaging without optical crosstalk. In addition, the multi-spectral imaging experiments of the field of view and different targets are carried out. The imaging effect is shown in Figures 21F–K.

#### 5.1.5 Polarization imaging

Polarization compound eye imaging consists of a lens array and polarization analysis and imaging technology. This technique allows for capturing and analyzing polarization information to provide additional insights beyond conventional intensity-based images. Polarization imaging offers benefits in characterizing surface roughness, texture direction, surface tendencies, surface conductivity, material properties, chemical characteristics, and water content properties of objects. It particularly excels in identifying surface contours and orientations. Moreover, polarization imaging technology has distinct advantages in remote image acquisition, capturing fine details, and recognizing targets with camouflage in challenging environments.

**FIGURE 22:**
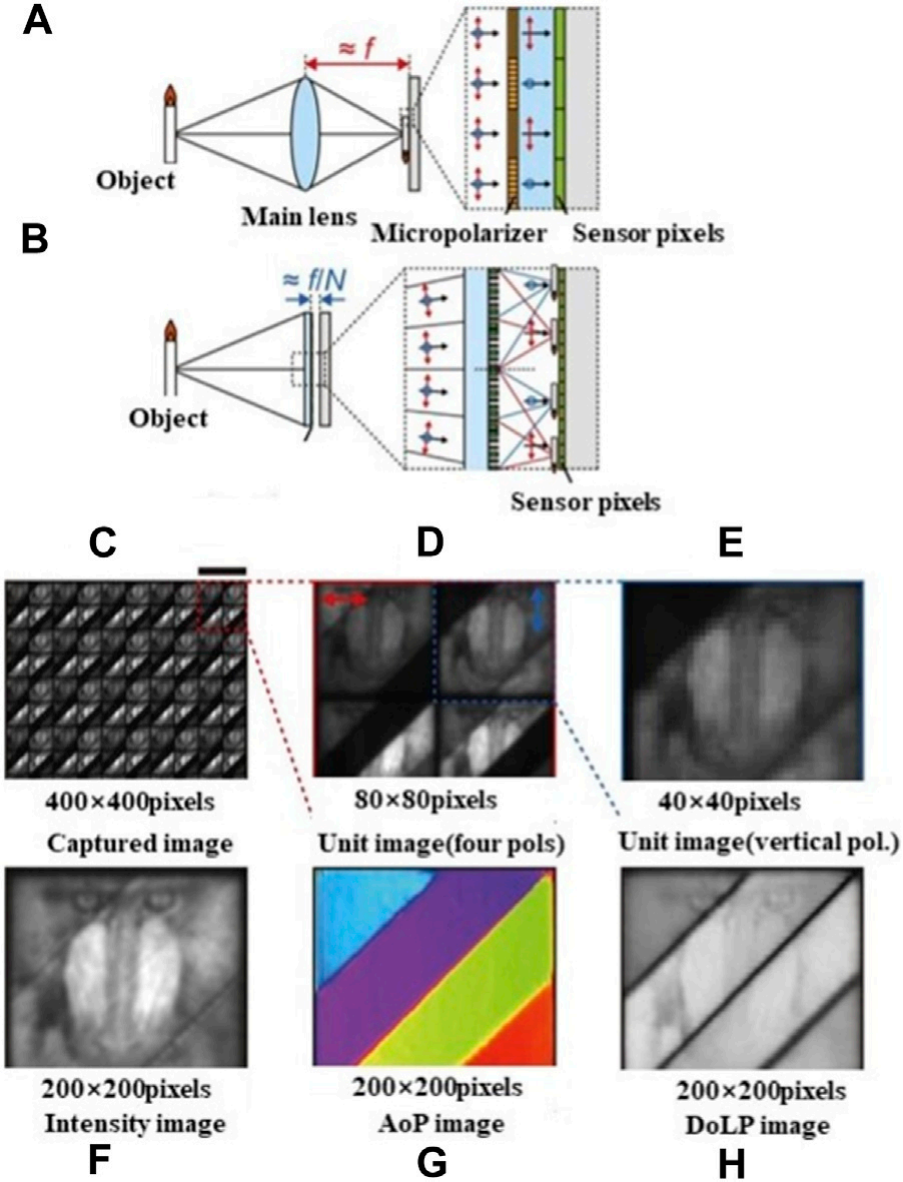
Polarized compound eye imaging. [Reproduced with permission from (Miyata et al., 2020)]. **(A)** Conventional focal plane division (DoFP) polarization camera. **(B)** Cross section of polarization camera based on metasurface. **(C–E)** composite compound eye imaging and magnification. **(F–H)** 200 × 200 pixel AoP and linear polarization (DoLP) images.

K. Kagawa et al. presented a polarization filter deep-focus compound eye camera for three-dimensional endoscopy Kagawa et al. (2012b). The camera incorporates polarization filters parallel and perpendicular to the polarized light in specific areas of the lens. Additionally, it employs wavefront coding (WFC) technology based on spherical aberration to achieve an extended depth of focus. A prototype called TOMBO, consisting of a 3 × 3 lens configuration and a 2.2 μm pixel color CMOS image sensor, was developed. Although the camera can achieve super-resolution image processing, its field of view (FOV) is limited. To address this, K. Kagawa et al. designed visible and near-infrared polarized compound eyes with a variable FOV by introducing fixed and moving small mirrors Kagawa et al. (2012c). Furthermore, M. Miyata et al. proposed a polarized compound eye camera with compound eye surface optics Miyata et al. (2020). The system consists of a standard image sensor and a metasurface layer. By eliminating the need for filters, the camera significantly reduces the required optical path length for imaging, surpassing the efficiency and size limitations of traditional polarization cameras. It achieves a 2-fold increase in the detected light and reduces the overall thickness to 1/10 of current polarization cameras. The camera exhibits exceptional sensitivity in imaging, with the imaging effect demonstrated in Figures 22C–H. Figures 22A, B depict the configuration of the proposed camera.

### 5.2 Outlook

Over the course of previous decades, significant advancements have been made in the research of artificial compound eyes pertaining to the design of their structures, fabrication techniques, and optical capabilities. Despite these successes, there is still significant room for improvement in artificial compound eyes. The prospects of four anticipated research focus on compound eye especially for curved compound eye are presented as follows.

1) Multi-material integrated manufacturing

Achieving a fully functional compound eye imaging system requires the replication of various components such as the cornea, crystalline cone, pigment cell, rhabdom, and retinula cell. Currently, research efforts are focusing on the precise manufacturing of compound eye lenses, which play a crucial role in image formation. However, to create a comprehensive and autonomous compound eye visual apparatus, it is necessary to integrate multiple components of the imaging system. This integration can be achieved through multi-material integrated manufacturing techniques. By utilizing this approach, different materials can be combined and fabricated together, resulting in a more seamless and efficient assembly of the compound eye imaging system. Multi-material integrated manufacturing allows for the precise placement and alignment of different components, reducing errors that may occur during subsequent assembly processes. This integration enhances the overall performance of the compound eye imaging system and improves its functionality as a whole. By replicating and integrating the various components accurately, researchers aim to create a fully functional, autonomous compound eye visual apparatus that can provide unique imaging capabilities, mimic the natural vision of insects, and find applications in fields like robotics, surveillance, and medical imaging.

Continued research and advancements in multi-material integrated manufacturing techniques will contribute to the development of more sophisticated compound eye imaging systems, bringing us closer to achieving a comprehensive and autonomous visual apparatus based on the principles of compound eyes.

2) Mass production of complex 3D compound eyes

The current mass production of compound eyes relies heavily on mold technology, which has limited three-dimensional controllability. This can restrict the level of precision and flexibility in manufacturing the compound eyes. To address this limitation, advanced 3D forming technologies for compound eyes with higher three-dimensional controllability are still required. These technologies aim to provide more precise and flexible manufacturing processes for compound eyes, allowing for improved functionalities and performance. By utilizing advanced 3D forming technologies, manufacturers can enhance the controllability of the compound eye’s three-dimensional structure, leading to better replication of the natural optical properties and functionalities of compound eyes.

This has significant industrial values in applications such as imaging systems, optical sensors, and other fields where the unique capabilities of compound eyes are desired. Continued research and development in this area can potentially drive advancements in manufacturing techniques, enabling the mass production of compound eyes with higher precision, performance, and industrial applicability.

3) High-stability material compound eye manufacturing technology

The current micro-compound eye structures made primarily of organic or organic-inorganic hybrid materials have indeed shown excellent performance. However, to overcome the limitations in corrosion resistance, heat resistance, mechanical strength, and high damage threshold, the utilization of high stability materials in manufacturing technology is of utmost importance. High stability materials offer enhanced properties in terms of corrosion resistance, heat resistance, mechanical strength, and high damage threshold, which are critical for the practical application of micro-compound eyes in engineering fields. These materials, such as advanced ceramics, engineered alloys, or innovative polymer composites, can provide the necessary durability and stability required for demanding applications. By incorporating high stability materials into the manufacturing process of micro-compound eyes, their performance and applications can be significantly improved. Researchers and engineers are actively exploring and developing new materials and manufacturing techniques to enhance the properties of micro-compound eyes and expand their potential uses in various engineering applications.

The pursuit of manufacturing technology using high stability materials is indeed essential for advancing the development and integration of micro-compound eyes in practical engineering applications. Continued research, collaboration, and innovation in this area will ultimately enable us to overcome the existing limitations and fully unlock the potential of micro-compound eyes.

4) Miniaturized high-resolution curved sensors

Commercially available photoelectric sensors are primarily planar sensors. However, the development of miniaturized high-resolution curved surface sensors has the potential to bring about significant improvements in various performance aspects of compound eye imaging systems. Curved surface sensors, also known as curved image sensors, are designed to mimic the structure of natural compound eyes found in insects and some other organisms. These sensors feature a curved shape that allows for a larger field of view and improved image resolution compared to traditional planar sensors. The main advantage of curved surface sensors is that each pixel on the sensor is oriented towards the center of the curved surface. This means that light rays from different angles can be more accurately captured by the individual pixels, resulting in improved imaging accuracy and a higher resolution across the entire field of view. The curved shape also reduces image distortion and glare, leading to better image quality. In addition to improved resolution and imaging accuracy, curved surface sensors can also offer faster response speeds. By curving the sensor, the distance between the lens and the pixels can be reduced, resulting in shorter optical paths and faster image capture.

Overall, the development of miniaturized high-resolution curved surface sensors has the potential to revolutionize imaging technology by significantly improving the performance of compound eye imaging systems. These sensors can enhance resolution, imaging accuracy, response speed, and other crucial aspects, opening up new possibilities in various industries such as robotics, medical imaging, and surveillance.

## 6 Conclusion

This paper provides a comprehensive review of microfabrication technologies, photoelectric detection, and applications of miniature compound eyes. The paper begins with an introduction to the structural composition and imaging principles of compound eyes, highlighting the similarities and distinctions. Next, it discusses the microfabrication technologies used for both planar and curved compound eyes, comparing their advantages and disadvantages. The paper then reviews the microfabrication approaches for photoelectric detection in different types of curved artificial compound eyes. Afterward, it lists the existing and potential functional applications of miniature compound eyes. Finally, the future development of compound eyes is discussed, focusing on areas such as multi-material integrated manufacturing technologies, mass production technologies for complex 3D compound eyes, and advancements in high-stability materials and miniaturized high-resolution curved sensors.

In conclusion, compound eyes have significant potential and unique advantages. However, further advancements in materials, microfabrication technologies, and detection are necessary to fully realize their practical applications in various fields.
